# Prediction of acute kidney injury after cardiac surgery: model development using a Chinese electronic health record dataset

**DOI:** 10.1186/s12967-022-03351-5

**Published:** 2022-04-09

**Authors:** Hang Zhang, Zhongtian Wang, Yingdan Tang, Xin Chen, Dongfang You, Yaqian Wu, Min Yu, Wen Chen, Yang Zhao, Xin Chen

**Affiliations:** 1 Department of Thoracic and Cardiovascular Surgery, Nanjing First Hospital, Nanjing Medical University, No. 68 Changle Road, Nanjing, 210006 China; 2 Department of Biostatistics, School of Public Health, Nanjing Medical University, No. 101 Longmian Avenue, Nanjing, 211166 China; 3 Department of Cardiovascular Surgery, Shanghai General Hospital, Shanghai Jiao Tong University School of Medicine, No. 100 Haining Road, Shanghai, 200080 China; 4 The Center of Biomedical Big Data and the Laboratory of Biomedical Big Data, Nanjing Medical University, No. 101 Longmian Avenue, Nanjing, 211166 China

**Keywords:** Acute kidney injury, Cardiac surgery, Machine learning, XGBoost, Random forest, Deep forest, Nomogram

## Abstract

**Background:**

Acute kidney injury (AKI) is a major complication following cardiac surgery that substantially increases morbidity and mortality. Current diagnostic guidelines based on elevated serum creatinine and/or the presence of oliguria potentially delay its diagnosis. We presented a series of models for predicting AKI after cardiac surgery based on electronic health record data.

**Methods:**

We enrolled 1457 adult patients who underwent cardiac surgery at Nanjing First Hospital from January 2017 to June 2019. 193 clinical features, including demographic characteristics, comorbidities and hospital evaluation, laboratory test, medication, and surgical information, were available for each patient. The number of important variables was determined using the sliding windows sequential forward feature selection technique (SWSFS). The following model development methods were introduced: extreme gradient boosting (XGBoost), random forest (RF), deep forest (DF), and logistic regression. Model performance was accessed using the area under the receiver operating characteristic curve (AUROC). We additionally applied SHapley Additive exPlanation (SHAP) values to explain the RF model. AKI was defined according to Kidney Disease Improving Global Outcomes guidelines.

**Results:**

In the discovery set, SWSFS identified 16 important variables. The top 5 variables in the RF importance matrix plot were central venous pressure, intraoperative urine output, hemoglobin, serum potassium, and lactic dehydrogenase. In the validation set, the DF model exhibited the highest AUROC (0.881, 95% confidence interval [CI] 0.831–0.930), followed by RF (0.872, 95% CI 0.820–0.923) and XGBoost (0.857, 95% CI 0.802–0.912). A nomogram model was constructed based on intraoperative longitudinal features, achieving an AUROC of 0.824 (95% CI 0.763–0.885) in the validation set. The SHAP values successfully illustrated the positive or negative contribution of the 16 variables attributed to the output of the RF model and the individual variable’s effect on model prediction.

**Conclusions:**

Our study identified 16 important predictors and provided a series of prediction models to enhance risk stratification of AKI after cardiac surgery. These novel predictors might aid in choosing proper preventive and therapeutic strategies in the perioperative management of AKI patients.

**Supplementary Information:**

The online version contains supplementary material available at 10.1186/s12967-022-03351-5.

## Background

Acute kidney injury (AKI), a common and potentially life-threatening clinical syndrome, is more and more frequent with increasing cardiac surgical volume in developed and developing countries. A meta-analysis reported an incidence of cardiac surgery-associated acute kidney injury (CSA-AKI) of 26.0–28.5% [1]. AKI not only severely affects in-hospital morbidity and mortality but also long-term prognosis. Patients who survive an episode of AKI after surgery are also at elevated risk of developing major adverse cardiovascular events, advanced chronic kidney disease, and all-cause death [2].

The current consensus definition of AKI depends on the increase of serum creatinine (Scr) and/or the presence of oliguria; however, this can lead to delayed diagnosis and treatment. Consequently, substantial efforts have been made to explore biomarkers or develop clinical prediction models in recent years. Several novel biomarkers were proposed to substitute Scr in the assessment of kidney function, such as NGAL, KIM-1, and DKK3 [3, 4]. However, an individual biomarker is inadequate in predicting AKI; that is to say, the pathophysiology of CSA-AKI is multifactorial and intricate. On the other hand, these biomarkers are costly and difficult to assay and are thus out of consideration by the majority of clinicians [5]. Clinical scoring systems (e.g., Cleveland Clinic score, Simplified Renal Index score, Mehta score, etc.) have been introduced into clinical practice for more than a decade [6–8], while widespread adoption of these models would be challenging. First, these models are developed following the traditional logistic regression method. Their derivation requires the statistical assumption regarding a linear relationship between covariates and outcomes, with analytical model restricting to selection of a small set of parameters that are known to be clinically relevant. Second, most of these models perform traditional feature selection methods based on a small scale of exposure variables and identify common risk factors (e.g., age, diabetes mellitus, hypertension, cardiac function, surgery type, etc.). These risk factors reflect preoperative conditions and generally present inadequate predictive power in different races or populations [9].

Recent advancements in electronic health record (EHR) systems, data accessibility, and artificial intelligence have raised great interest in developing completely electronic data-driven machine learning (ML) models for predicting specific clinical outcomes. Several studies have used ML to predict inpatient AKI using EHR data. For instance, a continuous deep learning algorithm can predict 55.8% of all inpatient episodes of AKI up to 48 h in advance and over 90% of all AKI patients requiring subsequent renal replacement therapy (RRT) [10]. Therefore, our first objective was herein to develop three tree-based ML models to predict CSA-AKI by incorporating preoperative, intraoperative, and early postoperative data from the EHR of Nanjing First Hospital. In addition, to uncover the “black-box” of ML, SHapley Additive exPlanation (SHAP) values were utilized to explain the ML model and evaluated individual variable prediction [11]. Given the generalizability of the linear model, a nomogram model was finally constructed based on the logistic regression analysis.

## Method

### Study design and participants

This is a retrospective, observational study. Consecutive patients who underwent cardiac surgery, admitted between January 2017 and June 2019, were recruited from Nanjing First Hospital. We enrolled patients who had received coronary artery bypass grafting (CABG), valve surgery, and a combination of both treatments. Patients were excluded if they met the following criteria: (i) aged < 18 years; (ii) preoperative AKI, end-stage renal disease, or dialysis; (iii) did not receive cardiopulmonary bypass (CPB); (iv) missing Scr data. Patient informed consent in this study was waived due to the retrospective nature of the study. This study was approved by the Ethical Committee of Nanjing First Hospital. We reported our work following TRIPOD statement guideline [12].

### Data preprocessing

The study cohort was acquired from two population-based databases, consisting of patient information available from EHR in digital format: Jiangsu Province Coronary Artery Bypass Grafting Register (218.2.200.37:2356/Multicenter) and Patient Information Management Platform (218.2.200.37:2356/PatientList). The databases are specifically designed for cardiac patients and consist of perioperative clinical characteristics including patient demographics, admission assessment, comorbidities, laboratory test, medication, surgical information, and CPB data. All clinical data regarding preoperative, intraoperative and early postoperative variables were included for model derivation (preoperative laboratory biomarkers were collected at 6 a.m. the next day following hospital admission; early postoperative variables were measured within 6 h after surgery). Missing values were filled in by a second manual review of the EHR, and personal information was de-identified before delivering for analysis. The final dataset was randomly partitioned to a discovery (80% of observations) set and a validation (20%) set.

### Anesthesia, CPB, and critical care

All participants received general intravenous-inhalation combined anesthesia, which was maintained intraoperatively with a continuous infusion of propofol (4–6 mg/kg/h), remifentanil (0.2–0.4 μg/kg/min), cisatracurium (0.2–0.3 mg/kg/h) as well as an intermittent addition of sufentanil and midazolam. CPB was performed with non-pulsatile perfusion, with a perfusion flow of 2.0–2.8 L/kg/min and a mean arterial pressure of 55–85 mmHg in most cases. During CPB, monitoring records included nasopharyngeal temperature, bladder temperature, rectal temperature, perfusion flow, oxygen delivery, perfusion pressure, central venous pressure (CVP), conventional ultrafiltration (CUF), and urine output. After the surgery was completed, patients were transferred to the intensive care unit (ICU) and placed on ventilators in synchronized intermittent mandatory ventilation or assist-control models set at 8–10 mL/kg tidal volume and 5 cmH_2_O positive end-expiratory pressure. Arterial blood gas was checked at the time of ICU admission; other laboratory measurements (e.g., blood cell analysis, liver and kidney function, coagulation function, etc.) were obtained within 6 h postoperatively.

### AKI definition

Postoperative AKI was defined according to the Scr-based criteria from the Kidney Disease: Improving Global Outcomes (KDIGO) consensus definition, specifically an acute increase in Scr ≥ 50% within 7 days or ≥ 0.3 mg/dL within 48 h compared with the baseline level, or a requirement for RRT [13]. In this case, the patient’s baseline Scr was determined by the Scr level measured at hospital admission.

### Model development

This study mainly comprised two stages: (1) feature selection; and (2) model development. We used a ML-based feature selection method, the sliding windows sequential forward feature selection (SWSFS), to identify the number of important variables among the 193 clinical features. Then two types of models were developed based on the selected variables including three tree-based supervised learning models and a nomogram model.

### Feature selection

First, we applied a random forest (RF) classifier to calculate variable importance score (VIS) according to the Gini index via the *importance* function in R. To minimize random errors, we performed the RF 30 times by setting the random seeds from 1 to 30 and calculated the average Gini index of each variable. SWSFS was used to identify a set of important variables. Briefly, VIS of all the clinical features (except age and gender) was obtained from RF and ranked by the averaged Gini index in descending order. Next, the features were included one by one to the RF model based on their VIS ranks. Afterward, we plotted the model error, which measured the “out of bag (OOB)” rate of each RF model consisting of different numbers of variables. Finally, the number of features was identified based on the lowest model error rate.

### Tree-based ensemble algorithms

We developed the prediction models using the following tree ensemble methods, which are the most popular and advanced ML algorithms for binary classification: extreme gradient boosting (XGBoost), RF, and deep forest (DF). Both XGBoost and RF use rules to binary split data based on decision trees. Generally, a tree with many splits will probably lead to overfitting and result in poor performance in new datasets. RF works based on the idea of the ensemble method, which collects individual decision trees, bagging and random feature selection, thus providing more accurate results and making the model more resistant to overfitting [14]. XGBoost is the engineering realization of the Gradient Boosting Decision Tree, which provides superior prediction by combining multiple decision trees in boosting ways [15]. As a novel and advanced deep learning method, DF generates a multi-layer cascade forest containing various RFs [16]. This structure has been designed to ensure the diversity of the model by including different types of forests. Each layer in the cascade forest receives the information processed by the previous stage and outputs the processing results to the next layer (Additional file 1: Fig. S1).

The area under the receiver operating characteristic curve (AUROC) was performed to compare the discrimination of various ML models. Model calibration was evaluated using a calibration plot based on the isotonic regression method [17]. Furthermore, for evaluating ML models, a series of interpretable parameters were determined: accuracy, sensitivity, specificity, positive predictive value, negative predictive value, and F score. It is worth noting that the correct interpretability of a ML model is challenging. We used SHapley Additive exPlanation (SHAP) values to explain the RF model. SHAP values are developed based on the concept of Shapley values from cooperative game theory [18]. It approximates a complex model to a linear model and evaluates variable importance to demonstrate the amount by which a given feature changes the prediction. Specificity, the SHAP values not only highlight the contributions of the individual variable to the model but also demonstrate the influence of each variable on global model effects. In addition, we used SHAP plot function to uncover the complex relationship between variables and outcomes in the RF model.

### Nomogram construction

We further constructed a nomogram model based on the variables selected by SWSFS. It is a logistic regression model. Any multicollinearity of the variables was excluded by establishing the variance inflation factor (VIF); the maximum VIF was 1.41 (Additional file 1: Fig. S2). Given the high association between AKI and procedure-related factors, the important intraoperative longitudinal data were handled as group-based trajectory modelling (GBTM). This approach is designed to identify clusters of individuals following similar progressions over time. We used the Stata command, *traj*, a plugin based on SAS PROC TRAJ macro, to fit a semi-parameter model for longitudinal data by maximum likelihood estimation [19]. The optimal number of groups with at least 5% of patients in the smallest trajectory was determined by establishing the Bayesian information criterion. Subsequently, the trajectory pattern of longitudinal data and remaining important features were incorporated into the multivariate logistic regression analysis to generate a nomogram model. This nomogram model included dynamic intraoperative information and provided a dynamic prediction paradigm. The discrimination of the nomogram was accessed using C-statistic, an index equivalent to AUROC. Model calibration was evaluated using the Brier score and visualized with a calibration plot using the 1000 bootstrap resampling method. A lower Brier score indicates superior calibration [20]. The clinical net benefit of the nomogram was estimated by decision curve analysis [21].

### Statistical analysis

For descriptive analyses, continuous variables are described as means (standard deviation) and categorical variables as frequencies with proportions. The clinical characteristics of patients who developed AKI or did not were compared using the Student’s *t* test, Mann Whitney *U* test, chi-square test, or Fisher’s exact probability method as appropriate. After a second manual review for data integrity, the dataset had missing data ranging from 0% to 1.37%. We handled the missing values by using the multiple imputation method. Statistical analyses were performed using Stata (version 13.0) with the package of *traj*, R (version 4.0.3) with packages of *mice*, *rms*, and *rmda*, and Python (version 3.8) with the packages of *sklearn*, *deep-forest*, and *shap*. A two-sided *P* value < 0.05 was considered statistically significant.

## Results

### Study population

From January 2017 to June 2019, 1457 consecutive patients were enrolled in the final cohort (Additional file 1: Fig. S3). The mean (standard deviation) of their age was 60 (12.3) years, and 848 (58.2%) of the participants were male. Of them, 486 (33.4%) underwent CABG, 802 (55.0%) were subjected to valve surgery, and 169 (11.6%) received concomitant CABG and valve surgery. Each individual patient had 193 clinical features. They were randomly assigned to a discovery set (n = 1170) or a validation set (n = 287). The rates of AKI were 24.3% and 24.0% in the discovery and validation sets, respectively. In both sets, differences in clinical characteristics between patients who developed AKI and those who did not are outlined in Additional file 1: Table S1.

### Feature selection

**Fig. 1:**
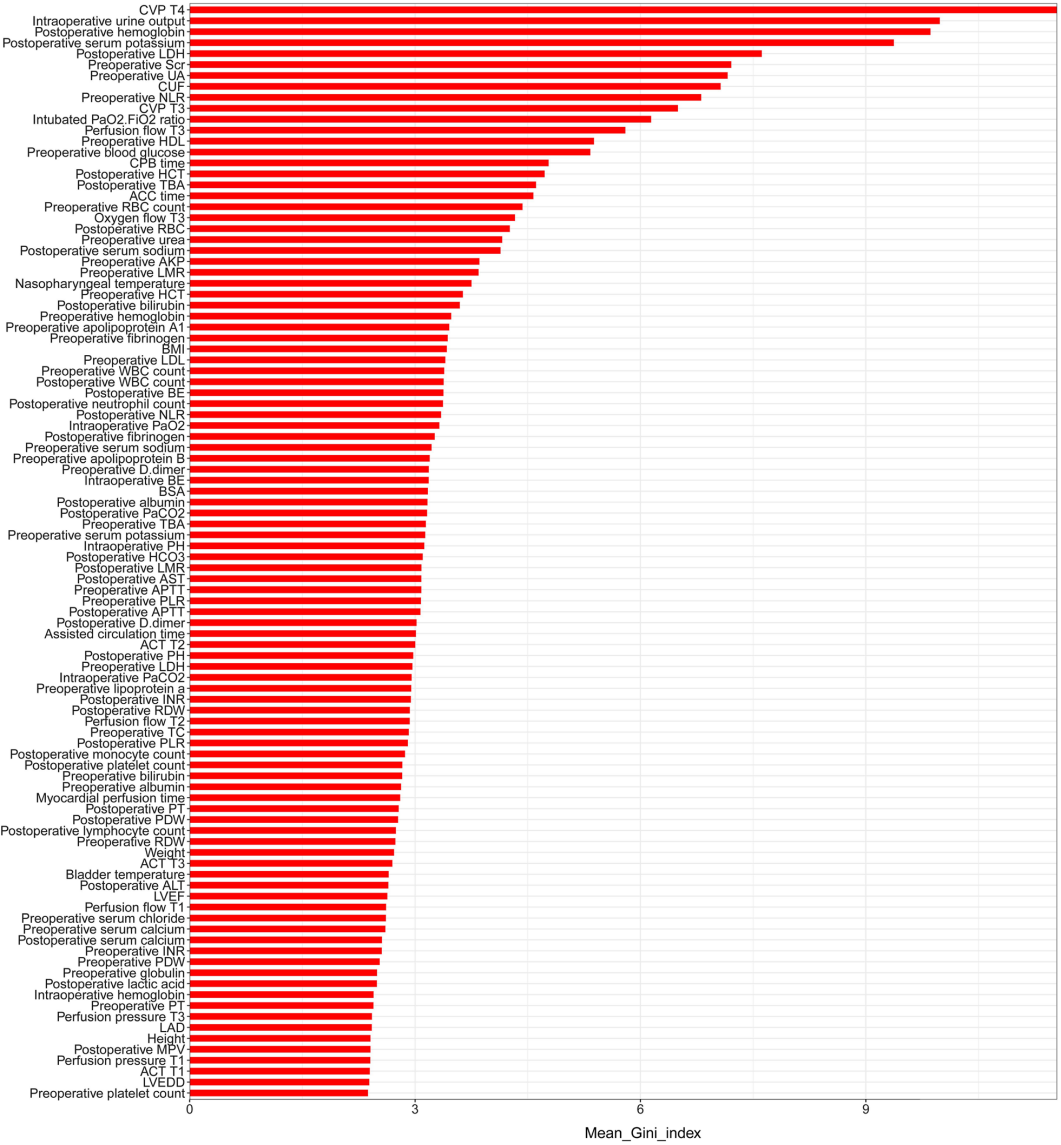
Importance matrix plot of random forest algorithm based on the discovery set

**Fig. 2:**
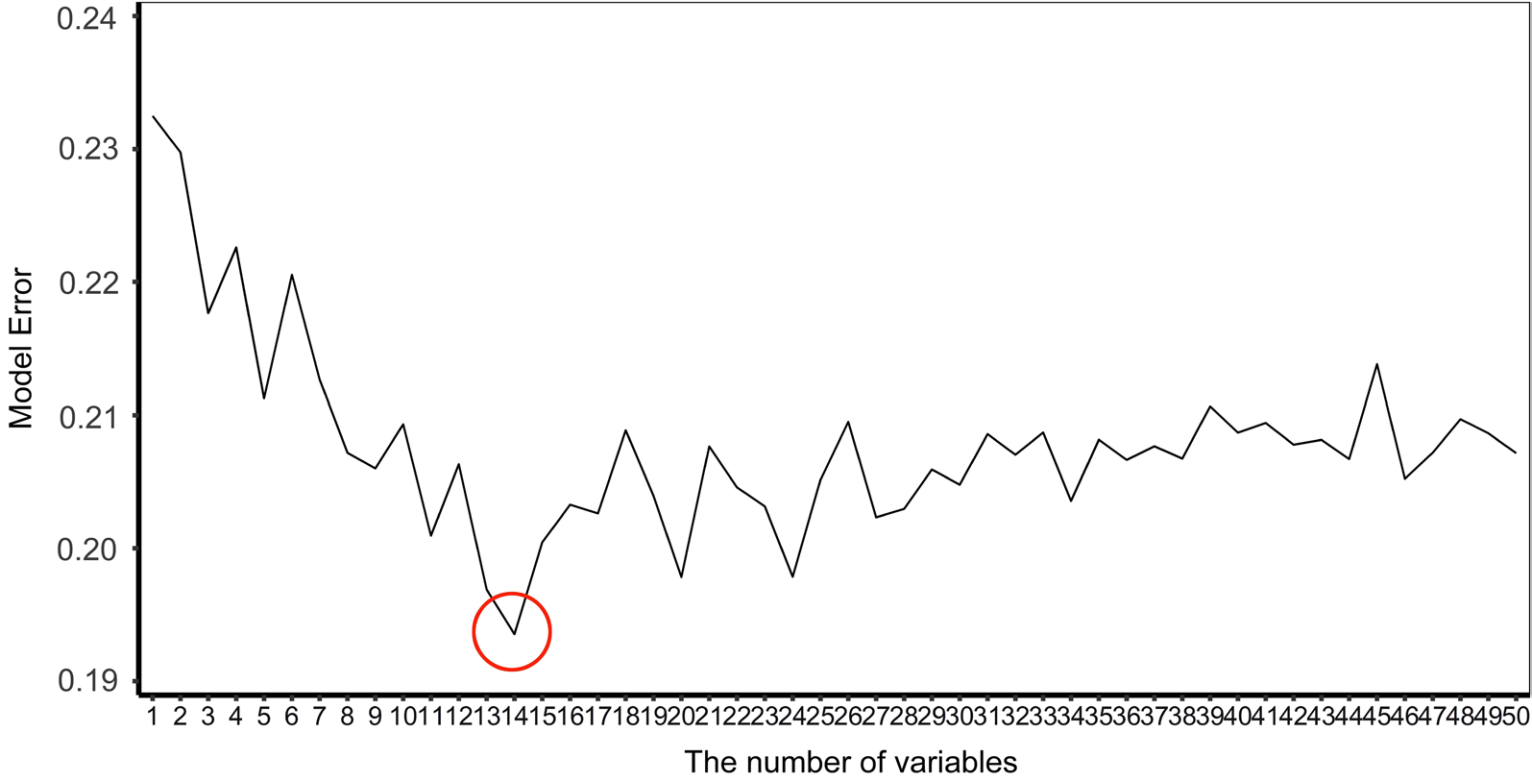
Feature selection by the sliding windows sequential forward feature selection method. First, variable importance score (VIS) of features was obtained from a random forest algorithm and ranked in descending order. The variables were then included one by one to the random forest model in order of VIS rank. Finally, the optimal number of variables (14 variables) was determined by minimum model error (red circle)

The VIS of each variable was obtained from the RF algorithm and ranked in descending order. Figure 1 illustrates the importance matrix plot of the top 100 features. Afterward, all features (except age and gender) were included in SWSFS one by one in order of their VIS ranks. Based on the lowest OOB error rate (Fig. 2), SWSFS identified 14 important features including five preoperative factors (Scr, neutrophil to lymphocyte ratio [NLR], blood glucose, uric acid [UA], and high-density lipoprotein [HDL]), five intraoperative factors (urine output, ultrafiltration volume, CVP_T3, CVP_T4, and perfusion flow_T3), and four early postoperative factors (intubated PaO_2_/FiO_2_ ratio, hemoglobin, serum potassium, and lactic dehydrogenase [LDH]).

### Tree-based learning models

**Fig. 3:**
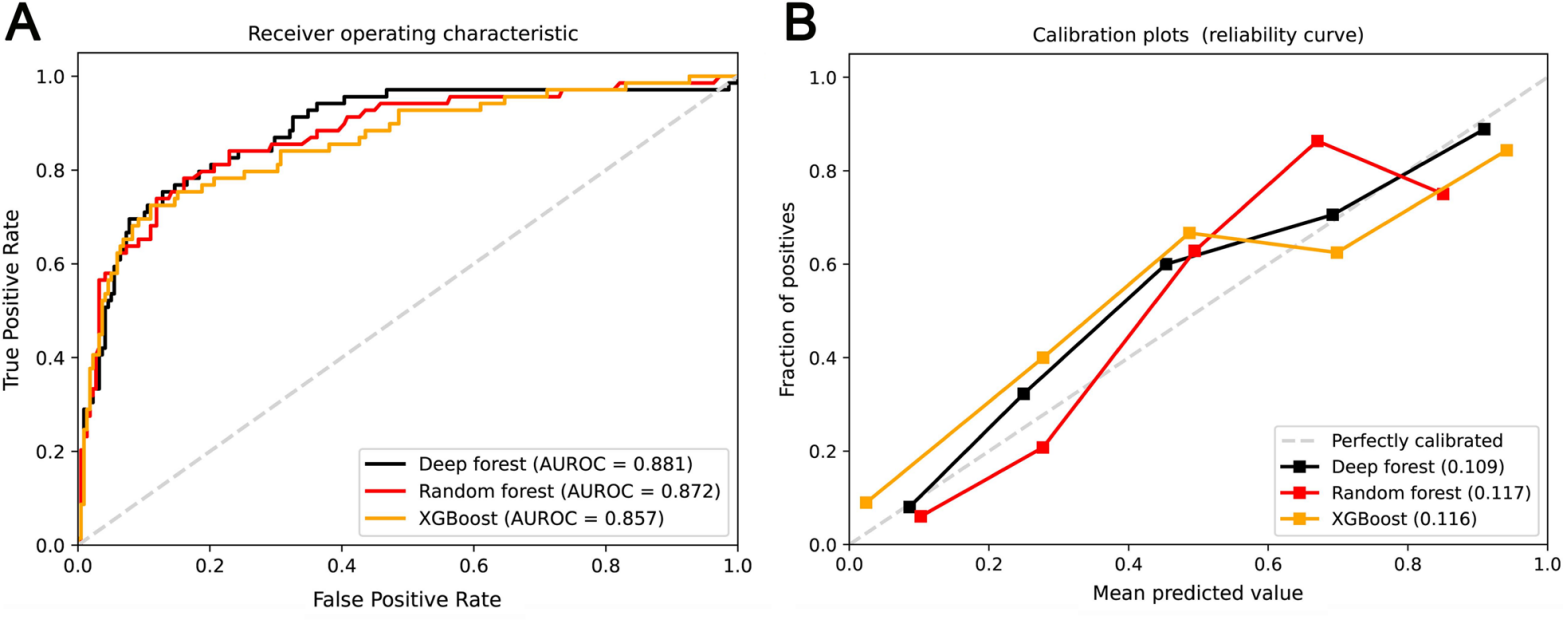
Performance of machine learning models. (**A**) Comparison of area under the receiver operating characteristic curves among XGBoost, random forest, and deep forest models in the validation set; (**B**) Calibration plots of XGBoost, random forest, and deep forest models in the validation set. The Brier score is reported in the lower right legend (the smaller the value, the better the calibration). AUROC: area under the receiver operating characteristic curve

Two covariates (age and gender) and the 14 important variables were included in the following ML models: XGBoost, RF, and DF. Figure 3A illustrates the model performance in the validation set, measured by AUROC. The DF model exhibited the largest AUROC (0.881, 95% confidence interval [CI] 0.831–0.930), followed by the RF model (0.872, 95% CI 0.820–0.923) and XGBoost model (0.857, 95% CI 0.802–0.912). Accordingly, five-fold cross-validation results of AUROC and accuracy of XGBoost, RF, and DF are summarized in Additional file 1: Table S2. The calibration plot demonstrated that all these models had well calibrated. The Brier scores were 0.109, 0.117, and 0.116 for the DF, RF, and XGBoost models, respectively (Fig. 3B). The parameters of these algorithms are presented in Additional file 1: Table S3.

### SHAP values through visualization

**Fig. 4:**
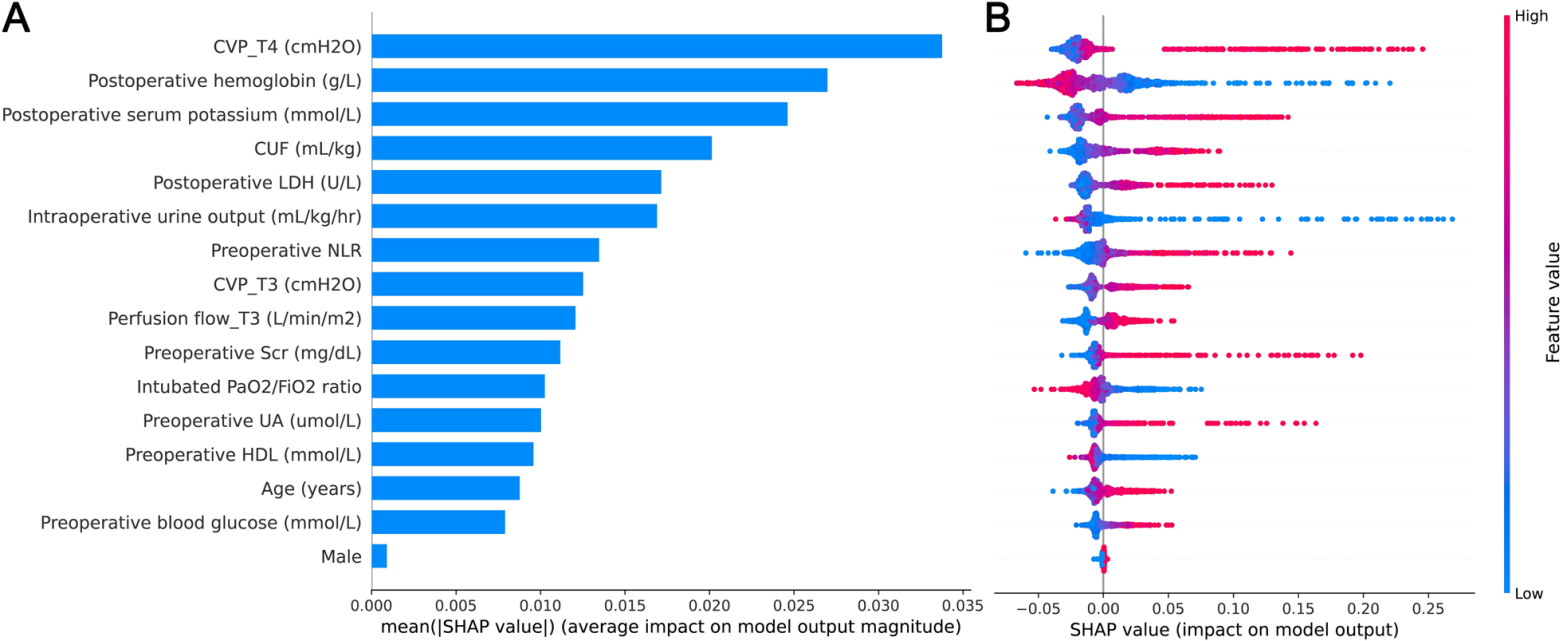
SHAP summary plot of the random forest model. (**A**) Average absolute impact of variables on the final model output magnitude ordered by decreasing feature importance; (**B**) The plot depicts the dot estimation on the model output of random forest model. Each dot represents an individual patient from the dataset. Red represents higher SHAP value of specific features; blue represents lower SHAP value of specific features. The higher the SHAP values, the greater the risk of developing acute kidney injury development. CVP: central venous pressure; CUF: conventional ultrafiltration; LDH: lactic dehydrogenase; NLR: neutrophil to lymphocyte ratio; Scr: serum creatinine; UA: uric acid; HDL: high-density lipoprotein

**Fig. 5:**
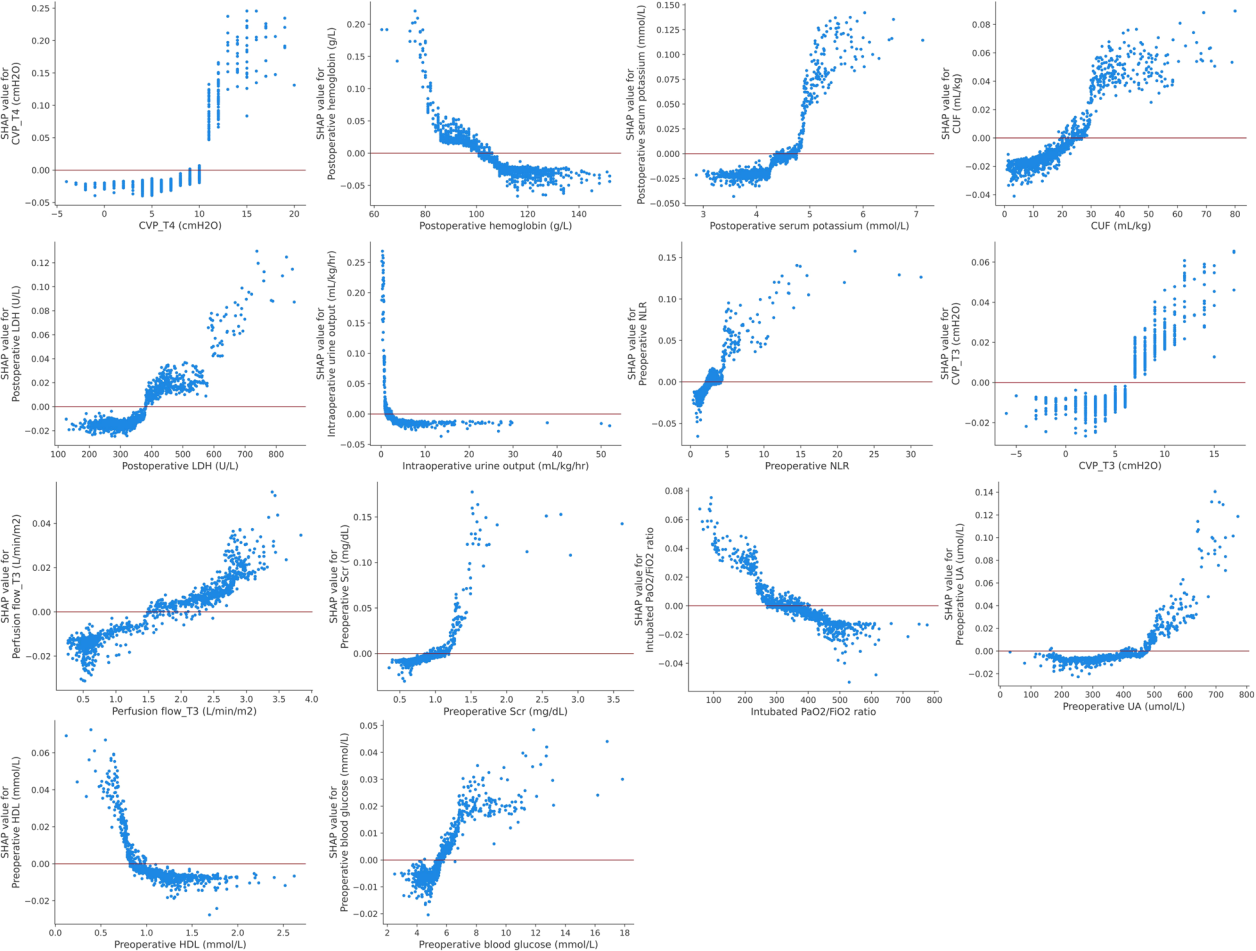
SHAP dependence plot of the random forest model. Each panel demonstrates that each feature affects the output of the random forest prediction model. The x-axis represents the raw values of each feature and the y-axis indicates the SHAP values of features. When the SHAP value of a specific feature exceeds zero, it indicates an increased risk of acute renal injury. CVP: central venous pressure; CUF: conventional ultrafiltration; LDH: lactic dehydrogenase; NLR: neutrophil to lymphocyte ratio; Scr: serum creatinine; UA: uric acid; HDL: high-density lipoprotein

**Fig. 6:**
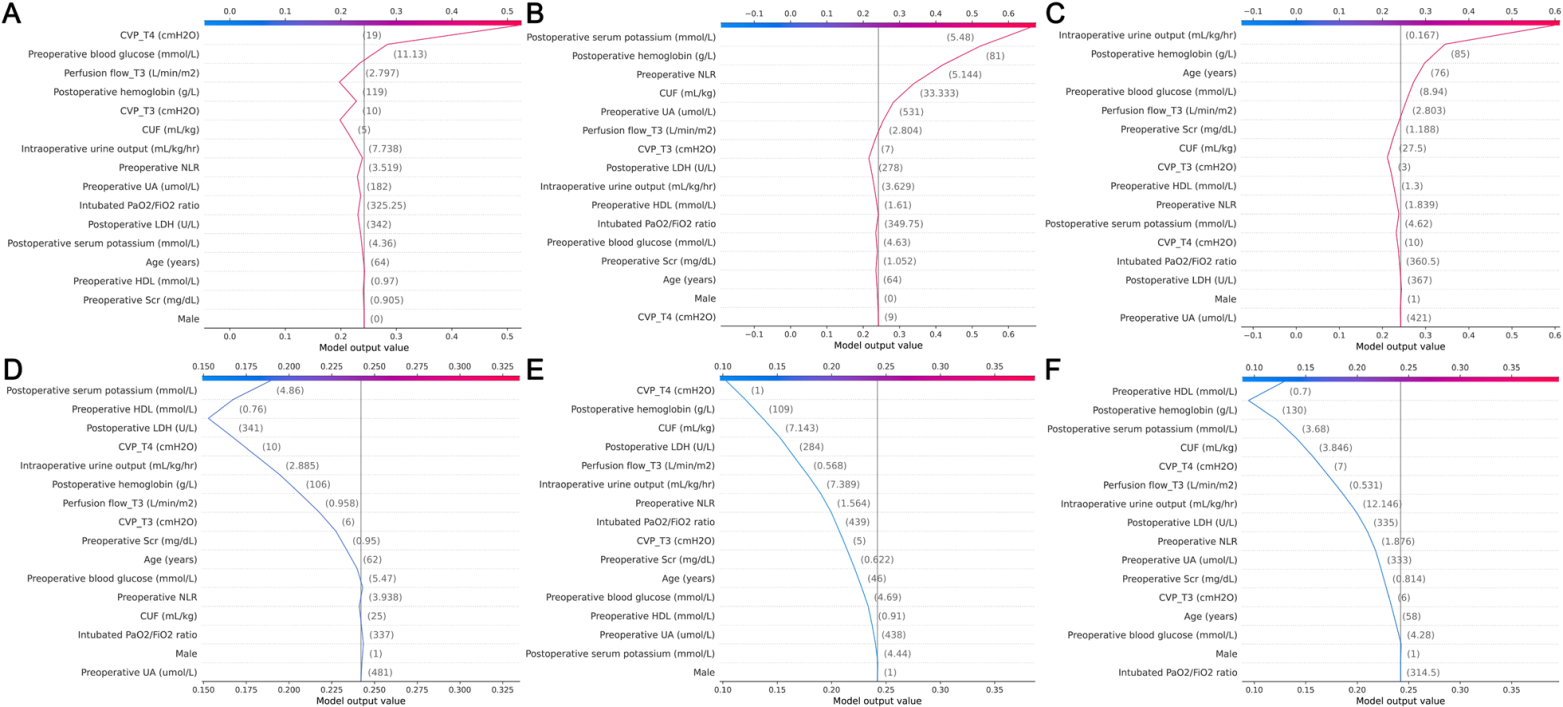
SHAP decision plot of individual patient-level prediction. The plots depict the decision path for predicting acute renal injury. All patients started with mean predictive values and were evaluated at each factor level to obtain the final probability of acute kidney injury. **A**–**C** Delineate illustrative examples of patients predicted to be acute renal injury; **D**–**F** display illustrative examples of patients predicted to be non-acute renal injury. CVP, central venous pressure; CUF: conventional ultrafiltration; LDH: lactic dehydrogenase; NLR: neutrophil to lymphocyte ratio; Scr: serum creatinine; UA: uric acid; HDL: high-density lipoprotein

We used SHAP values to provide accurate attribution values for each variable within the RF model. Figure 4A describes the SHAP summary plot for each input variable in the discovery set. The y-axis displays the 16 variables ranked in order of importance with their mean absolute SHAP values. The x-axis indicates the SHAP values associated with each variable and patient, which allowed the determination of whether a feature had a negative effect on the prediction toward a non-AKI class or positive effect on the prediction toward an AKI class. (Fig. 4B). Besides, SHAP dependent plot provides a visualization of the impact for individual observations. Using zero as a dividing line, a feature’s effect can be clearly observed regarding its positive or negative contribution to the model (Fig. 5). The individual patient-level prediction was depicted using the SHAP decision plot. This plot function allows for a better understanding of the individual decision path and describes why patients A-C were predicted as AKI whereas patients D-F were not (Fig. 6).

### Group-based trajectory modelling and nomogram model

**Fig. 7:**
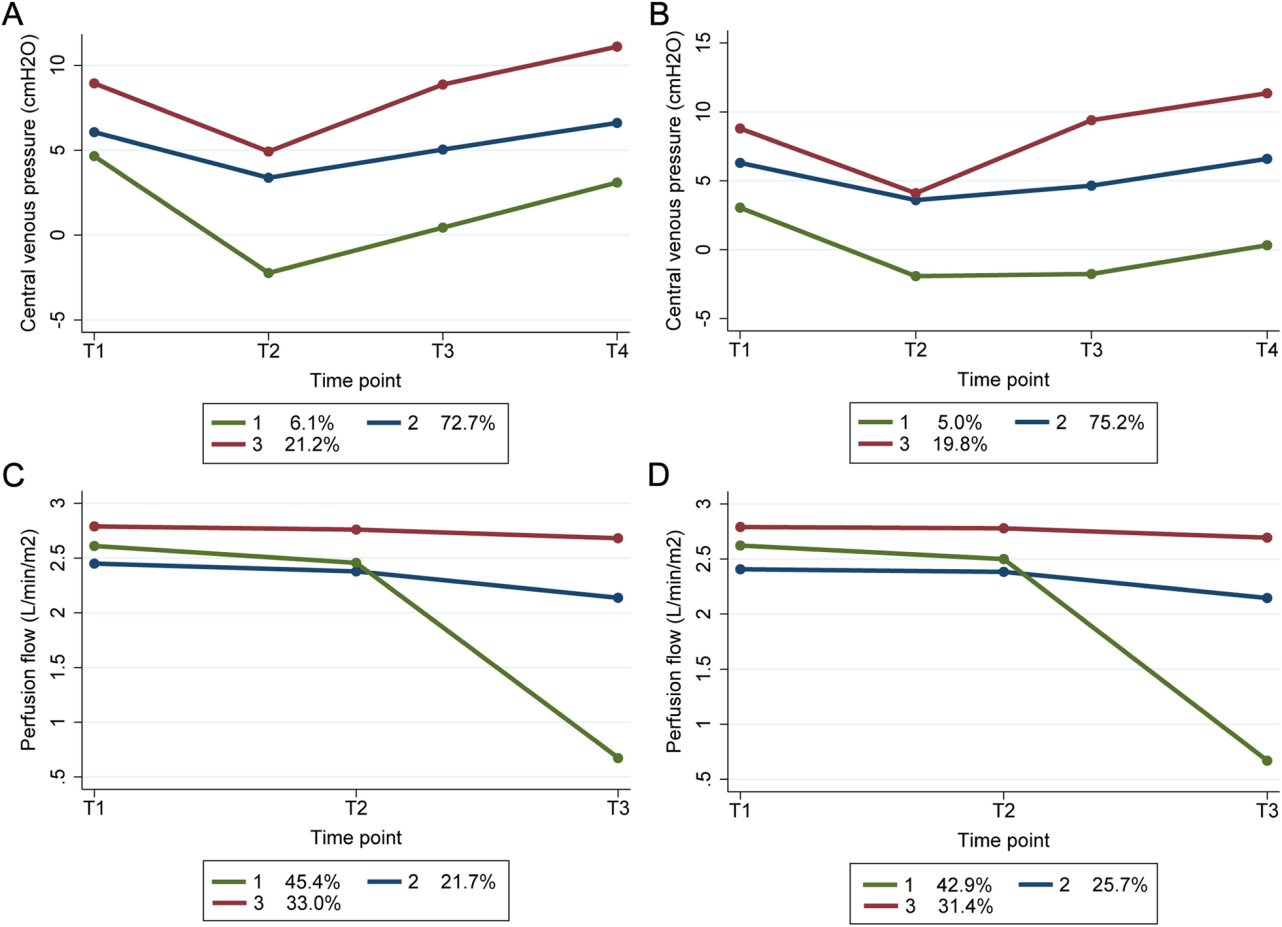
Group-based trajectory modelling of central venous pressure and perfusion flow in the discovery (**A**) and validation (**B**) sets. These variables were repeatedly measured at specific time points (T1, initial phase of cardiopulmonary bypass; T2, 20 min after aortic cross-clamping; T3, 10 min after aortic declamping; T4, before weaning off from cardiopulmonary bypass)

**Fig. 8:**
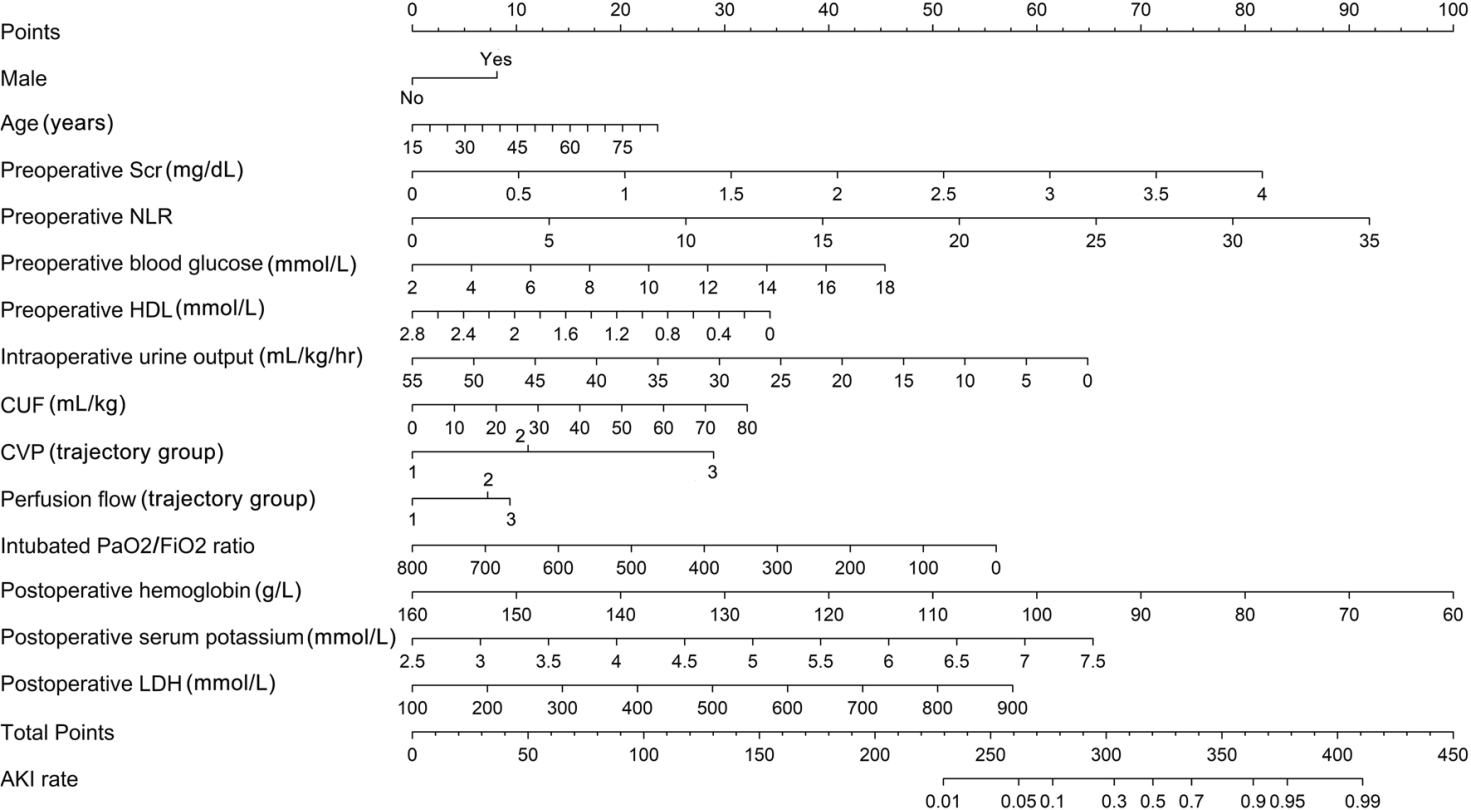
Nomogram predicting acute kidney injury after cardiac surgery. Scr: serum creatinine; NLR: neutrophil to lymphocyte ratio; HDL: high density lipoprotein; CUF: conventional ultrafiltration; CVP: central venous pressure; LDH: lactic dehydrogenase; AKI: acute kidney injury

**Fig. 9:**
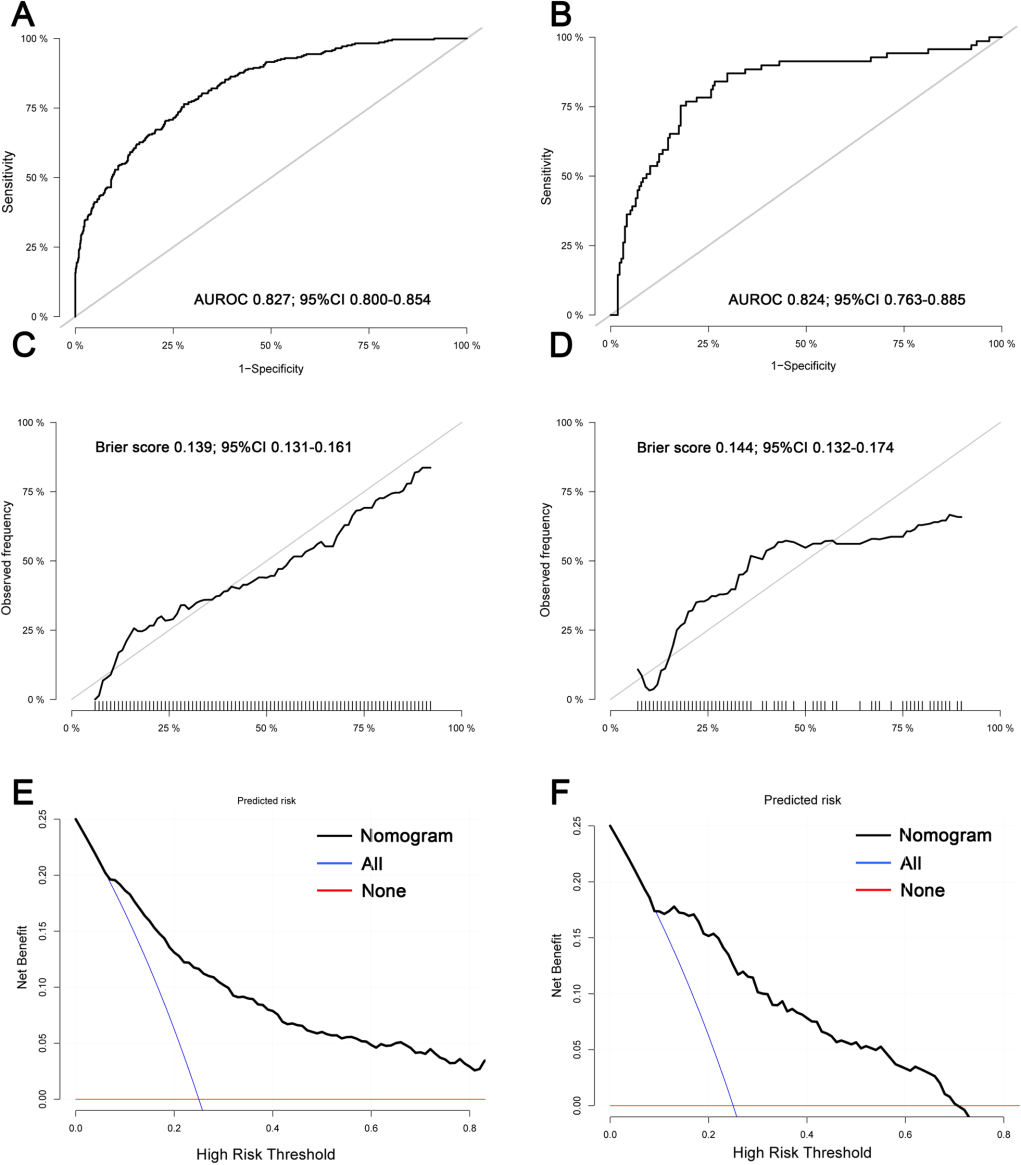
Performance evaluation of the nomogram model. Receiver operating characteristic curve of the nomogram in the discovery (**A**) and validation (**B**) sets; 1000-resample bootstrapped calibration plot of the nomogram in the discovery (**C**) and validation (**D**) sets; decision curve analysis of the nomogram in the discovery (**E**) and validation (**F**) sets. AUROC, area under the receiver operating characteristic curve; CI: confidence interval

Among the 16 important predictors, CVP_T3, CVP_T4, and perfusion flow_T3 are CPB-related factors associated with AKI development. To observe their dynamic pattern, GBTMs were created in both sets, in which the three clusters of CVP and perfusion flow following different progressions could be observed. The higher indices the trajectory groups of CVP and perfusion flow, the greater the risk of AKI development (Fig. 7). Inclusion of trajectory groups and other important variables in the multivariate logistic regression model resulted in 14 predictors (excluding UA, *P* > 0.1) that were statistically significant for AKI (Table 1). These independently associated risk factors were incorporated to form an AKI estimation nomogram (Fig. 8). The nomogram was internally validated using the bootstrap validation method. The nomogram achieved good discrimination in the discovery set, with a C-statistic of 0.827 (95% CI 0.800–0.854) and a bootstrap-adjusted C-statistic of 0.810. Correspondingly, in the validation set, the nomogram displayed a C-statistic of 0.824 (95% CI 0.763–0.885). In both sets, 1000 bootstrap resampling calibration plots confirmed an optimal agreement between the predicted and observed risk of AKI (Fig. 9A–D). Furthermore, the decision curve analysis revealed that the nomogram could provide clinical net benefit for most of the examined probabilities (Fig. 9E, F).

**Table 1:** Multivariate logistic regression model for predicting acute kidney injury after cardiac surgery based on discovery set

Risk factor	β	OR (95% CI)	*P* value
Age, years	0.0170	1.017 (1.001–1.035)	0.048
Male	0.4076	1.503 (1.027–2.209)	0.036
Preoperative Scr, mg/dL	1.0381	2.824 (1.427–5.859)	0.004
Preoperative NLR	0.1333	1.143 (1.065–1.233)	< 0.001
Preoperative blood glucose, mmol/L	0.1441	1.155 (1.048–1.273)	0.003
Preoperative HDL, mmol/L	-0.6207	0.537 (0.299–0.947)	0.034
Intraoperative urine output, mL/kg/hr	-0.0602	0.942 (0.889–0.990)	0.027
CUF, mL/kg	0.0205	1.021 (1.009–1.033)	< 0.001
CVP, cmH_2_O (trajectory group)			
2 vs. 1	0.5632	1.756 (0.778–4.427)	0.199
3 vs. 1	1.4666	4.334 (1.838–11.314)	0.001
Perfusion flow, L/min/m^2^ (trajectory group)			
2 vs. 1	0.3693	1.447 (0.937–2.227)	0.094
3 vs. 1	0.4780	1.613 (1.091–2.389)	0.016
Intubated PaO_2_/FiO_2_ ratio	− 0.0035	0.996 (0.995–0.998)	< 0.001
Postoperative hemoglobin, g/L	− 0.0505	0.951 (0.938–0.963)	< 0.001
Postoperative serum potassium, mmol/L	0.6649	1.944 (1.448–2.623)	< 0.001
Postoperative LDH, U/L	0.0037	1.005 (1.003–1.006)	< 0.001
Intercept	− 2.8021		

OR, odds ratio; CI, confidence interval; Scr, serum creatinine; NLR, neutrophil to lymphocyte ratio; HDL, high density lipoprotein; CUF, conventional ultrafiltration; CVP, central venous pressure; LDH, lactic dehydrogenase

## Discussion

In this study, we applied the SWSFS technique to screen for clinical characteristics and developed a series of models to optimize AKI prediction following cardiac surgery. Using SWSFS, we identified 14 important risk factors associated with AKI among the 193 clinical variables, thus boosting efficiency by incorporating preoperative, intraoperative, and early postoperative data from EHR. The performance of the tree ensemble ML algorithms (XGBoost, RF, and DF) was clinically satisfactory, with AUROC ranging 0.857–0.881 in the validation set. In addition, we constructed a nomogram model for AKI which also presented good performance both in terms of discrimination (AUROC 0.824) and calibration (Brier score 0.144). Our study highlights the value of EHR data in the evaluation of AKI. These important perioperative factors might be helpful in providing individualized preventive strategies and delivering proper treatments in the management of AKI after cardiac surgery.

In addition to some well-known risk factors (e.g., age, gender, hypertension, diabetes mellitus, Scr, etc.) that have been identified by previous studies, most variables are novel predictors for AKI risk prediction. Intraoperative factors reflect acute physiological responses during surgery and play pivotal roles in the development of AKI, particularly the unique physiological perturbations of CPB. In this study, we generated developmental trajectories to describe the course of factors over time. Two specific time point measurements of CVP (T3 and T4) were found to be strong predictors, highlighting the effect of intraoperative venous congestion on renal function. Traditionally, CSA-AKI is considered to be caused by renal hypoperfusion due to hypotension, inadequate perfusion flow, or renal ischemia [22, 23]. This concept has been challenged by accumulating evidence that elevated CVP is a more powerful hemodynamic determinant than mean perfusion pressure for the development of postoperative AKI [24]. More recently, Lopez et al. [25] uncovered that higher levels of CVP during cardiac surgery were independently associated with higher odds of AKI. They also demonstrated that venous congestion is more accurate than hypotension in predicting AKI. CVP-induced AKI can therefore be regarded as “congestive kidney failure”. When examining the trajectory pattern of perfusion flow, the separate clusters of perfusion flow at T3 measurement in our trajectory analysis implied that some patients (high-level cluster) might go through cardiac insufficiency, hemodynamic instability, or other unstable intraoperative conditions after aortic declamping and require additional mechanical assistance before weaning off from CPB. The positive effect of ultrafiltration volume on AKI risk may be another latent indicator of the congestive state of the body, as CUF is used for fluid removal to reduce fluid overload [26]. Taken together, these findings demonstrate that not only renal ischemia but also renal congestion plays a vital role in worsening kidney function. Our study suggested that the clinician may need to pay more attention to hemodynamic changes, in particular during the cardiac resuscitation period. Although adequate urine output does not assure normal kidney function as a result of non-pulsatile flow and cold-induced diuresis, the presence of oliguria typically indicates an acute response to renal hypoperfusion. This result is consistent with the study by Tseng et al. [27], who observed that intraoperative urine output was the most influential feature in predicting AKI.

Early postoperative laboratory biomarkers could reflect the acute pathophysiology of kidney injury. In this study, we identified four laboratory biomarkers (intubated PaO_2_/FiO_2_ ratio, serum potassium, hemoglobin, and LDH) associated with CSA-AKI. These biomarkers reflect the patient’s overall disease severity. For example, hypoxemia (low intubated PaO_2_/FiO_2_ ratio) is a severe complication after cardiac surgery and has been shown to be highly related to prolonging mechanical ventilation, respiratory complications, and in-hospital death [28]. The loss in glomerular filtration rate reserve usually leads to electrolyte disorder. It was observed that some patients experienced abrupt deterioration in renal function and decreased glomerular filtration capacity in the early postoperative period, resulting in fluid overload and elevated serum potassium levels [29]. LDH is abundant in the kidney, heart, liver, and muscle and is, therefore, most commonly measured to detect tissue damage as well as disease severity of critical patients [30]. Although LDH acts as a nonspecific biomarker for kidney injury, it demonstrates adequate predictive value for AKI risk prediction in several clinical settings [31, 32]. On the other hand, the elevated LDH in the immediate postoperative period may be an indicator of hemolysis from CPB, and CPB-induced hemolysis is associated with the development of AKI [33].

Previous studies have concluded that inflammation, oxidative stress, and endothelial dysfunction are central components of the pathogenesis of AKI. NLR, a promising marker of inflammation, has been identified as a novel predictor of AKI [34]. Moreover, it has emerged as a potential biomarker for lethal outcomes and adverse events in patients undergoing cardiac surgery [35, 36]. Interestingly, lower baseline HDL levels were independently associated with an increased risk of AKI after cardiac surgery. This relationship was also observed in Smith et al.’s study [37]. Systematically, high HDL levels inhibit systemic inflammation and reduce oxidative stress via acting as receptors of prooxidant lipids and associated antioxidant enzymes and therefore play a role in the pathogenesis of AKI [37–39]. However, the natural effect of HDL may be pharmacologically modified by traditional preoperative lipid-lowering treatment in cardiac patients; novel pharmacologic agents with the potential for improving HDL function are warranted. Collectively, the pathophysiology of CSA-AKI is complex and multifactorial. Our study identified a set of important factors attributed to CSA-AKI such as venous congestion, renal hypoperfusion, inflammatory response, metabolic disorder, and baseline renal function. However, many of these factors are modifiable. Using these factors may therefore provide a basis for early diagnosis, prevention, and treatment strategies in the preoperative, intraoperative, and early postoperative management of AKI patients, such as optimizing hemodynamic status, intensified early postoperative biomarkers monitoring, and individualized blood glucose and lipids management.

Before implementing ML models in clinical practice, their predictive power must be validated in different clinical settings. Previous studies developed ML models using all features as input variables [27, 40]. However, inclusion of numerous features makes the models much more complicated and difficult to validate in additional representative datasets, as most variables are irrelevant to AKI classification. Besides, many ML algorithms exhibit a decrease in predictive power when the number of variables is significantly higher than optimal [41]. For example, as outlined in Additional file 1: Fig. S4, these ML models were further evaluated with all variables as input variables, and no improvement in model discrimination was noted. Our study confirms the significance of feature selection in ML applications. We applied SWSFS technique to determine the optimal and minimum size of features, thus increasing the efficiency and usefulness of the models for further validation.

Our study has several strengths. First, a major barrier to the widespread use of ML models is their correct interpretation, as a true “black-box” can hardly be accepted by clinicians or decision-makers. As an additive model explainable approach, SHAP analysis is seldom employed in ML application. We utilized the SHAP values to demystify the ML, providing a “white-box” AKI prediction model that allowed a quick comprehension of the effect of a single feature on the model’s prediction. This explainable or “white-box” predictive technique may be helpful in ML transportability across hospitals. Second, the three tree-based ensemble learning algorithms demonstrated high calculating efficiency and may be adapted to certain medical working environments. Indeed, XGBoost and RF have the advantages of being trained quickly and providing reliable feature importance estimates and are increasingly emphasized as competitive alternatives to traditional regression methods. In addition, both XGBoost and RF algorithms are bootstrapping method applications, which can improve predictive power when available datasets are small. Notably, for the first time to our knowledge, we used DF to predict AKI after cardiac surgery. As an alternative to the deep learning framework, DF improves the robustness of the traditional deep learning method working on small-scale clinical data and provides an effective solution for binary classification. Third, given the importance of intraoperative factors on AKI development, we applied GBTM method to handle intraoperative longitudinal data in which the dynamic process of variables over time could be clearly observed. GBTM identifies distinctive clusters of individual trajectories within the population, determining the subgroup patients at different risk levels; it provides a critical time point for clinical decision making. By monitoring intraoperative hemodynamic parameters, it is expected to build more accurate dynamic early warning systems that can help clinicians timely identify patients at risk of postoperative AKI. Fourth, the incidence of AKI in our study was generally in line with the report from a meta-analysis [1], indicating that our patient cohort is representative of cardiac patients in general. Finally, the dataset contained only a few missing data because most of the missing values were filled in during a second manual review of EHR. Therefore, the impact of missing data on the prediction models is negligible.

However, several limitations should also be considered. First, the models were developed based on the dataset derived from a single center with patients undergoing on-pump cardiac surgery. Consequently, before the models can be implemented in clinical practice for new patients, their predictive performance would require training and evaluation on other races, nationalities, or additional datasets. Second, postoperative urine output (less than 0.5 mL/kg/h for 6 h) was not used to define AKI due to its unavailability in the majority of patients. However, we were unlikely to miss vital clinical patients as urine output may have been maintained by diuretics. Moreover, due to intensified monitoring in ICU, persistent oliguria is uncommon and transient oliguria may simply imply insufficient volume resuscitation. Third, although SHAP values can explain most traditional ML models, they cannot explain the DF algorithm. In other words, the correct interpretation of DF remains challenging. We are working on developing a more advanced algorithm for DF that could provide variable importance estimation. Fourth, we did not include traditional scoring systems to make model comparisons, because the most extensively used and robust models for AKI are those designed for AKI requiring RRT. Given the high incidence of subclinical AKI and its strong association with adverse outcomes, more advanced models should be developed to predict any-stage AKI after cardiac surgery.

Taken together, our study highlights the potential of tree-based ensemble methods in generating robust AKI prediction tools. By contrast to clinical scoring systems or biomarkers, ML algorithm is a completely data-driven prediction tool. As for clinical practice, a web-based online tool can be developed to facilitate the application of the ML model. By inputting the values of features of a patient, the online tool would estimate the probability of developing AKI. With advances in EHR, it can be easily transferred to the EHR system to calculate AKI risk by automatically reading features.

ML algorithms for clinical data analysis have revolutionized the traditional way of conducting cardiovascular research. As clinicians continue to gather significant amounts of patient data through EHR, more novel associations between specific features and AKI will be identified. Future work is ongoing on the development of more advanced ML algorithms and EHR systems for real-time adjustment of the AKI risk after cardiac surgery, which in return will optimize treatment and enhance prognosis.

## Conclusions

In this study, based on the 16 important perioperative predictors, we successfully established three tree-based ML models and a nomogram model to optimize CSA-AKI risk prediction.

## Supplementary Information

**Additional file 1:** **Figure S1.** Illustration of cascade forest structure. **Figure S2.** Variance expansion factor assessment of 14 variables included in the logistic regression model. **Figure S3.** Formation of the discovery and validation sets with acute kidney injury after cardiac surgery. **Figure S4.** Comparison of area under the receiver operating characteristic curves among the XGBoost, random forest, and deep forest models in the validation set. **Table S1.** Clinical characteristics of patients in the discovery and validation sets who did or did not develop acute kidney injury after cardiac surgery. **Table S2.** Five-fold cross-validation results of AUROC and accuracy of XGBoost, random forest, and deep forest model. **Table S3.** Classifiers’ predictive performance in the validation set.

## Data Availability

The original contributions presented in the study are included in the article and additional files. Further data that support the findings of this study are available from the corresponding author on reasonable request.

## Abbreviations

AKI: Acute kidney injury
CSA-AKI: Cardiac surgery-associated acute kidney injury
EHR: Electronic health record
SWSFS: Sliding windows sequential forward feature selection
XGBoost: Extreme gradient boosting
RF: Random forest
DF: Deep forest
AUROC: Area under the receiver operating characteristic curve
SHAP: SHapley Additive explanation
Scr: Serum creatinine
ML: Machine learning
RRT: Renal replacement therapy
CABG: Coronary artery bypass grafting
CPB: Cardiopulmonary bypass
ICU: Intensive care unit
VIS: Variable importance score
OOB: Out of bag
GBTM: Group-based trajectory modelling
CVP: Central venous pressure
CUF: Conventional ultrafiltration
NLR: Neutrophil to lymphocyte ratio
UA: Uric acid
HDL: High-density lipoprotein
LDH: Lactic dehydrogenase
