# Supplementary material for: Prediction of acute kidney injury after cardiac surgery: model development using a Chinese electronic health record dataset
Source: J Transl Med. 2022 Apr 9;20:166. doi: 10.1186/s12967-022-03351-5 (PMC8994277; doi:10.1186/s12967-022-03351-5)
Supplement: Supplementary file 1 — Additional file 1: Figure S1. Illustration of cascade forest structure. Figure S2. Variance expansion factor assessment of 14 variables included in the logistic regression model. Figure S3. Formation of the discovery and validation sets with acute kidney injury after cardiac surgery. Figure S4. Comparison of area under the receiver operating characteristic curves among the XGBoost, random forest, and deep forest models in the validation set. Table S1. Clinical characteristics of patients in the discovery and validation sets who did or did not develop acute kidney injury after cardiac surgery. Table S2. Five-fold cross-validation results of AUROC and accuracy of XGBoost, random forest, and deep forest model. Table S3. Classifiers’ predictive performance in the validation set. [file 12967_2022_3351_MOESM1_ESM.docx]

**Prediction of acute kidney injury after cardiac surgery: model development using a Chinese electronic health record dataset**

**Supplementary material**

**Figure S1.** Illustration of cascade forest structure.

**Figure S2.** Variance expansion factor assessment of 14 variables included in the logistic regression model.

**Figure S3.** Formation of the discovery and validation sets with acute kidney injury after cardiac surgery.

**Figure S4.** Comparison of area under the receiver operating characteristic curves among the XGBoost, random forest, and deep forest models in the validation set.

**Table S1.** Clinical characteristics of patients in the discovery and validation sets who did or did not develop acute kidney injury after cardiac surgery.

**Table S2.** Five-fold cross-validation results of AUROC and accuracy of XGBoost, random forest, and deep forest model.

**Table S3.** Classifiers’ predictive performance in the validation set.


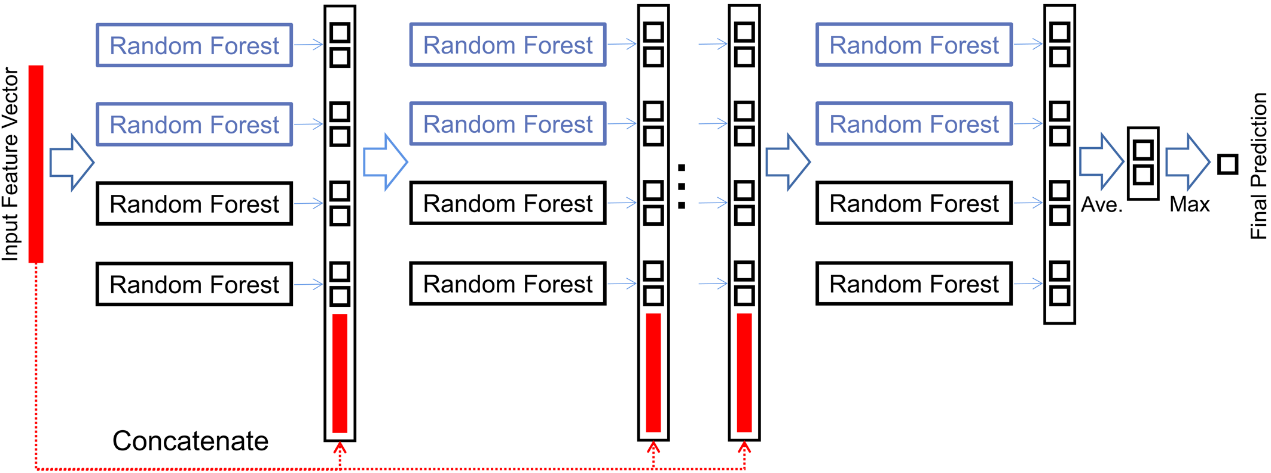


**Figure S1.** Illustration of cascade forest structure. Each level of the cascade consists of two random forests (light blue) and two completely random forests (black). Each forest outputs a two-dimensional class vector, which is then concatenated for representation of original input

**
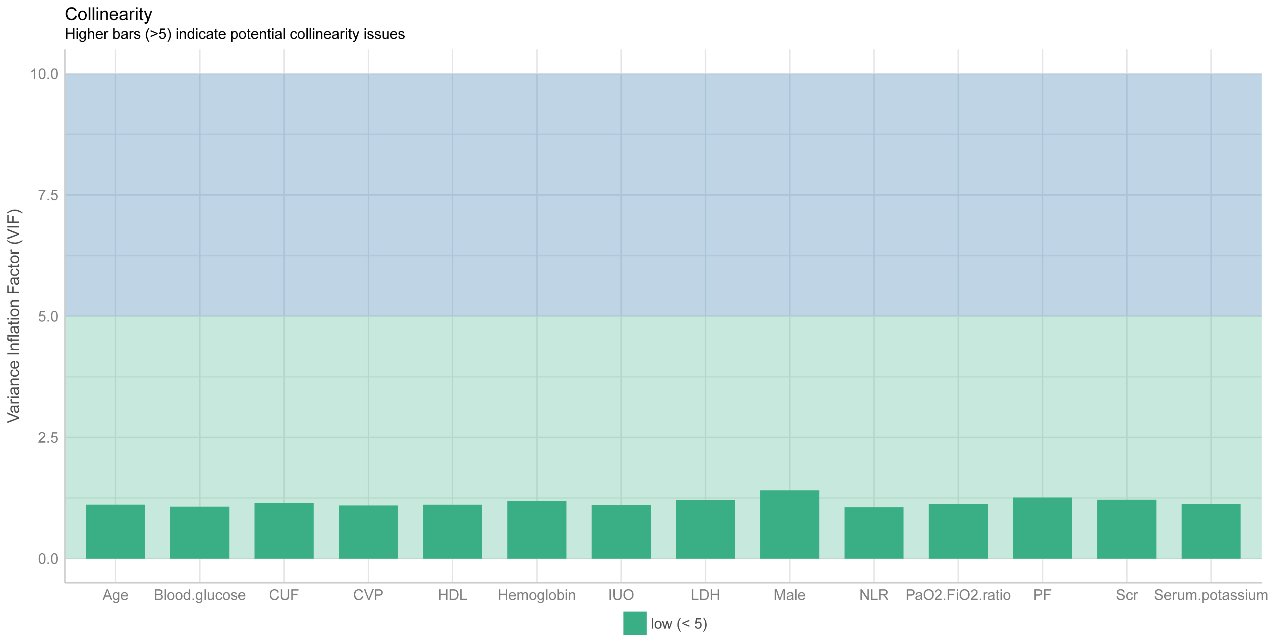
**

**Figure S2.** Variance expansion factor assessment of 14 variables included in the logistic regression model. Variance expansion factor less than 5 indicates no multicollinearity exists between variables. CUF, conventional ultrafiltration; CVP, central venous pressure; IUO, intraoperative urine output; LDH, lactic dehydrogenase; NLR, neutrophil to lymphocyte ratio; PF, perfusion flow; Scr, serum creatinine

**
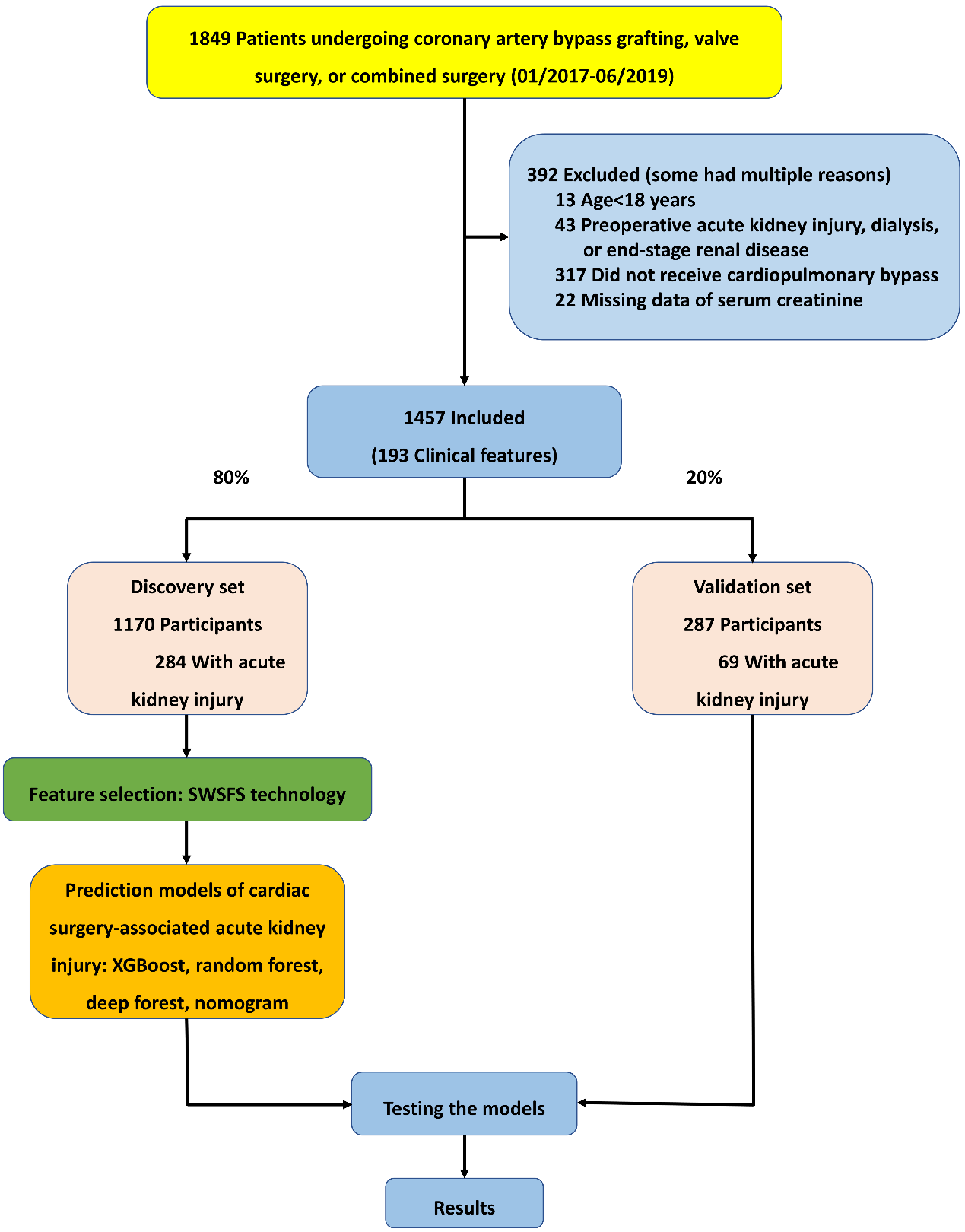
**

**Figure S3.** Formation of the discovery and validation sets with acute kidney injury after cardiac surgery

**
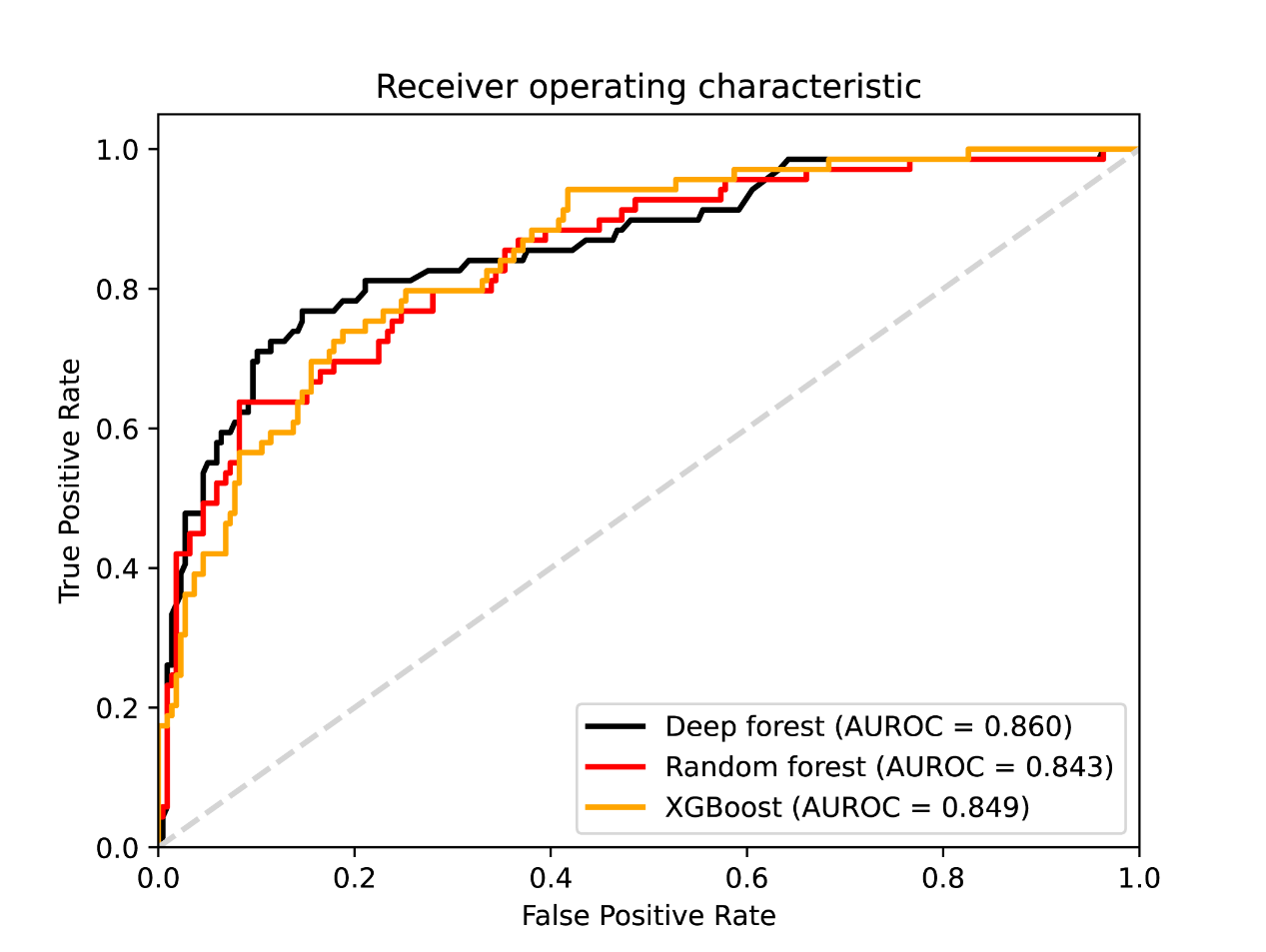
**

**Figure S4.** Comparison of area under the receiver operating characteristic curves among the XGBoost, random forest, and deep forest models in the validation set. The models included all features as input variables

**Table S1.** Clinical characteristics of patients in the discovery and validation sets who did or did not develop acute kidney injury after cardiac surgery

| **Clinical feature** | **Discovery set (n=1170)** | | **P-value** | **Validation set (n=287)** | | **P-value** |
| --- | --- | --- | --- | --- | --- | --- |
|  | **Non-AKI**  **(n=886)** | **AKI**  **(n=284)** |  | **Non-AKI**  **(n=218)** | **AKI**  **(n=69)** |  |
| **Demographic characteristics** | | | | | | |
| Age, years | 61.1 (10.4) | 64.5 (10.1) | <0.001 | 60.5 (10.4) | 65.2 (9.5) | 0.001 |
| Male | 496 (56.0%) | 186 (65.5%) | 0.006 | 121 (55.5%) | 45 (65.2%) | 0.199 |
| Rural area | 491 (55.4%) | 144 (50.7%) | 0.187 | 115 (52.8%) | 39 (56.5%) | 0.683 |
| Insurance | 473 (53.4%) | 167 (58.8%) | 0.127 | 123 (56.4%) | 39 (56.5%) | 1.000 |
| Height, cm | 164.0 (8.1) | 164.5 (8.4) | 0.362 | 164.4 (8.6) | 164.8 (7.9) | 0.735 |
| Weight, kg | 64.5 (11.1) | 65.8 (11.7) | 0.113 | 64.7 (11.0) | 65.5 (10.0) | 0.603 |
| BMI, kg/m^2^ | 23.9 (3.2) | 24.2 (3.3) | 0.183 | 23.9 (3.1) | 24.0 (2.8) | 0.643 |
| BSA, m^2^ | 1.7 (0.2) | 1.7 (0.2) | 0.128 | 1.7 (0.2) | 1.7 (0.2) | 0.615 |
| **Comorbidities and hospital evaluation** | | | | | | |
| Smoker | 93 (10.5%) | 38 (13.4%) | 0.218 | 15 (6.9%) | 9 (13.0%) | 0.173 |
| Diabetes mellitus |  |  | <0.001 |  |  | 0.001 |
| IDDM | 42 (4.7%) | 29 (10.2%) |  | 5 (2.3%) | 8 (11.6%) |  |
| NIDDM | 118 (13.3%) | 54 (19.0%) |  | 27 (12.4%) | 15 (21.7%) |  |
| No history | 726 (81.9%) | 201 (70.8%) |  | 186 (85.3%) | 46 (66.7%) |  |
| Hypertension | 464 (52.4%) | 180 (63.4%) | 0.001 | 101 (46.3%) | 38 (55.1%) | 0.259 |
| Hyperlipemia | 141 (15.9%) | 57 (20.1%) | 0.125 | 34 (15.6%) | 20 (29.0%) | 0.021 |
| COPD | 33 (3.7%) | 17 (6.0%) | 0.141 | 9 (4.1%) | 4 (5.8%) | 0.749 |
| PVD | 19 (2.1%) | 11 (3.9%) | 0.165 | 8 (3.7%) | 3 (4.3%) | 0.798 |
| CVA | 69 (7.8%) | 25 (8.8%) | 0.673 | 19 (8.7%) | 5 (7.2%) | 0.893 |
| Hepatic insufficiency | 17 (1.9%) | 9 (3.2%) | 0.311 | 6 (2.8%) | 2 (2.9%) | 1.000 |
| Hyperthyroidism | 7 (0.8%) | 1 (0.4%) | 0.687 | 2 (0.9%) | 2 (2.9%) | 0.240 |
| Hypothyroidism | 15 (1.7%) | 10 (3.5%) | 0.106 | 3 (1.4%) | 2 (2.9%) | 0.603 |
| Abnormal chest X-ray | 72 (8.1%) | 29 (10.2%) | 0.333 | 13 (6.0%) | 8 (11.6%) | 0.194 |
| IE | 20 (2.3%) | 7 (2.5%) | 1.000 | 4 (1.8%) | 1 (1.4%) | 1.000 |
| Hydrothorax | 20 (2.3%) | 10 (3.5%) | 0.339 | 3 (1.4%) | 5 (7.2%) | 0.020 |
| Angina | 378 (42.7%) | 138 (48.6%) | 0.093 | 84 (38.5%) | 37 (53.6%) | 0.038 |
| Previous MI | 130 (14.7%) | 55 (19.4%) | 0.073 | 34 (15.6%) | 20 (29.0%) | 0.021 |
| NYHA III-IV class | 255 (28.8%) | 120 (42.3%) | <0.001 | 46 (21.1%) | 32 (46.4%) | <0.001 |
| AF | 224 (25.3%) | 75 (26.4%) | 0.764 | 65 (29.8%) | 23 (33.3%) | 0.687 |
| Previous PCI | 59 (6.7%) | 23 (8.1%) | 0.488 | 11 (5.0%) | 5 (7.2%) | 0.563 |
| Allergy | 45 (5.1%) | 7 (2.5%) | 0.090 | 11 (5.0%) | 3 (4.3%) | 1.000 |
| CAS |  |  | 0.287 |  |  | 0.508 |
| Non | 787 (88.8%) | 244 (85.9%) |  | 197 (90.4%) | 60 (87.0%) |  |
| Unilateral | 37 (4.2%) | 12 (4.2%) |  | 8 (3.7%) | 5 (7.2%) |  |
| Bilateral | 62 (7.0%) | 28 (9.9%) |  | 13 (6.0%) | 4 (5.8%) |  |
| No diseased vessels |  |  | 0.090 |  |  | 0.165 |
| Non | 475 (53.6%) | 129 (45.4%) |  | 127 (58.3%) | 31 (44.9%) |  |
| 1 | 53 (6.0%) | 24 (8.5%) |  | 6 (2.8%) | 3 (4.3%) |  |
| 3 | 318 (35.9%) | 117 (41.2%) |  | 80 (36.7%) | 31 (44.9%) |  |
| 2 | 40 (4.5%) | 14 (4.9%) |  | 5 (2.3%) | 4 (5.8%) |  |
| Leftmain disease | 128 (14.4%) | 50 (17.6%) | 0.232 | 28 (12.8%) | 9 (13.0%) | 1.000 |
| Euroscore II, % | 5.4 (2.1) | 6.0 (2.4) | <0.001 | 5.2 (2.1) | 6.2 (2.5) | 0.004 |
| Previous surgery | 10 (1.1%) | 7 (2.5%) | 0.152 | 3 (1.4%) | 2 (2.9%) | 0.620 |
| **Laboratory test** | | | | | | |
| Scr, mg/dL | 0.8 (0.2) | 1.0 (0.4) | <0.001 | 0.8 (0.2) | 1.0 (0.5) | 0.001 |
| TC, mmol/L | 4.2 (1.1) | 4.1 (1.2) | 0.223 | 4.2 (1.1) | 4.1 (1.4) | 0.626 |
| LDL, mmol/L | 2.5 (0.9) | 2.5 (1.0) | 0.310 | 2.5 (0.9) | 2.5 (1.1) | 0.952 |
| Blood glucose, mmol/L | 5.4 (1.5) | 5.8 (1.8) | 0.001 | 5.2 (1.2) | 5.8 (2.0) | 0.030 |
| LDH, mmol/L | 210.8 (77.0) | 222.4 (82.6) | 0.037 | 206.1 (56.8) | 221.0 (93.6) | 0.212 |
| Albumin, g/L | 40.4 (3.8) | 39.6 (3.6) | 0.002 | 40.8 (4.0) | 39.2 (4.1) | 0.004 |
| Globulin, g/L | 27.6 (4.5) | 27.7 (4.5) | 0.640 | 27.9 (5.0) | 28.2 (5.7) | 0.679 |
| AKP, U/L | 73.9 (26.5) | 80.3 (32.8) | 0.003 | 70.6 (20.7) | 77.5 (28.3) | 0.064 |
| Bilirubin, umol/L | 13.7 (8.0) | 14.2 (9.2) | 0.421 | 13.9 (7.4) | 14.2 (9.5) | 0.831 |
| TBA, umol/L | 6.0 (6.9) | 6.6 (5.8) | 0.186 | 6.2 (6.1) | 7.7 (8.8) | 0.203 |
| Urea, mmol/L | 6.3 (2.1) | 7.2 (3.2) | <0.001 | 6.3 (2.0) | 7.8 (3.7) | 0.001 |
| UA, umol/L | 353.4 (103.7) | 396.5 (133.5) | <0.001 | 345.1 (92.6) | 393.3 (137.8) | 0.008 |
| Serum potassium, mmol/L | 4.0 (0.4) | 4.0 (0.4) | 0.084 | 4.0 (0.4) | 4.0 (0.5) | 0.240 |
| Serum sodium, mmol/L | 140.0 (2.4) | 139.9 (2.9) | 0.494 | 139.7 (2.7) | 139.4 (3.1) | 0.518 |
| Serum chlorine, mmol/L | 101.7 (3.2) | 101.8 (3.3) | 0.672 | 101.8 (3.4) | 101.3 (3.1) | 0.293 |
| Serum calcium, mmol/L | 2.3 (0.1) | 2.3 (0.1) | 0.023 | 2.3 (0.2) | 2.3 (0.2) | 0.314 |
| HDL, mmol/L | 1.1 (0.3) | 1.0 (0.3) | <0.001 | 1.1 (0.3) | 1.0 (0.3) | <0.001 |
| Apolipoprotein A1, g/L | 1.3 (0.3) | 1.2 (0.3) | 0.003 | 1.3 (0.3) | 1.2 (0.3) | 0.001 |
| Apolipoprotein B, g/L | 0.8 (0.2) | 0.8 (0.3) | 0.829 | 0.8 (0.3) | 0.8 (0.3) | 0.918 |
| Lipoprotein a, mg/L | 254.2 (247.1) | 253.1 (262.4) | 0.949 | 240.4 (228.1) | 258.7 (282.0) | 0.623 |
| Urine glucose | 0.1 (0.4) | 0.1 (0.5) | 0.213 | <0.1 (0.1) | 0.2 (0.6) | 0.034 |
| Urine protein | <0.1 (0.3) | 0.1 (0.4) | <0.001 | <0.1 (0.2) | 0.1 (0.3) | 0.102 |
| Urine WBC count | 37.5 (193.6) | 28.6 (120.0) | 0.356 | 25.8 (68.6) | 53.2 (176.4) | 0.210 |
| Urine RBC count | 75.3 (1395.0) | 88.0 (686.1) | 0.838 | 12.6 (71.6) | 6.3 (17.8) | 0.238 |
| WBC count (×10^^9^/L) | 6.3 (1.9) | 6.9 (2.3) | <0.001 | 6.2 (1.7) | 7.1 (2.8) | 0.020 |
| NLR | 2.4 (1.6) | 3.5 (3.6) | <0.001 | 2.3 (1.1) | 4.2 (4.3) | <0.001 |
| PLR | 115.0 (50.2) | 132.2 (128.2) | 0.028 | 113.4 (43.3) | 131.9 (103.8) | 0.154 |
| LMR | 6.1 (7.0) | 5.1 (3.8) | 0.002 | 6.3 (5.4) | 4.9 (3.4) | 0.016 |
| RBC count (×10^^12^/L) | 4.5 (0.6) | 4.3 (0.7) | <0.001 | 4.5 (0.5) | 4.3 (0.7) | 0.024 |
| Hemoglobin, g/L | 134.5 (17.0) | 130.1 (20.5) | 0.001 | 133.3 (16.5) | 126.8 (19.8) | 0.015 |
| HCT, % | 40.5 (4.7) | 39.3 (5.6) | 0.001 | 40.3 (4.5) | 38.0 (6.0) | 0.005 |
| RDW, % | 13.3 (1.3) | 13.7 (1.8) | 0.001 | 13.4 (1.6) | 13.7 (1.3) | 0.208 |
| Platelet count (×10^^9^/L) | 193.3 (62.0) | 191.5 (63.0) | 0.683 | 195.8 (62.4) | 190.0 (64.3) | 0.515 |
| MPV, fL | 11.3 (1.3) | 11.3 (1.2) | 0.720 | 11.3 (1.4) | 11.0 (1.3) | 0.189 |
| PDW, % | 14.1 (2.9) | 14.4 (2.7) | 0.141 | 14.0 (3.2) | 13.8 (2.7) | 0.714 |
| PT, sec | 11.8 (3.7) | 11.8 (3.1) | 0.926 | 11.9 (3.2) | 11.8 (2.7) | 0.924 |
| INR | 1.0 (0.3) | 1.0 (0.3) | 0.836 | 1.0 (0.3) | 1.0 (0.2) | 0.678 |
| APTT, sec | 28.1 (5.5) | 27.6 (6.0) | 0.141 | 28.0 (5.3) | 28.7 (4.7) | 0.316 |
| Fibrinogen, g/L | 3.0 (0.9) | 3.2 (1.6) | 0.007 | 2.9 (1.3) | 3.7 (2.1) | 0.010 |
| D-dimer, μg/mL | 0.6 (1.6) | 0.8 (1.6) | 0.032 | 0.6 (1.9) | 1.1 (2.7) | 0.177 |
| **Echocardiography findings** | | | | | | |
| LVEF, % | 59.3 (8.7) | 57.7 (9.3) | 0.012 | 60.5 (7.1) | 58.0 (8.9) | 0.041 |
| LVEDD, mm | 53.4 (8.5) | 54.6 (8.7) | 0.039 | 52.7 (8.0) | 52.9 (7.8) | 0.843 |
| LAD, mm | 48.0 (11.7) | 49.6 (10.0) | 0.021 | 48.1 (10.3) | 49.3 (9.1) | 0.375 |
| LVDd, mm | 404 (45.6%) | 129 (45.4%) | 1.000 | 95 (43.6%) | 35 (50.7%) | 0.368 |
| Pulmonary hypertension | 260 (29.3%) | 100 (35.2%) | 0.073 | 61 (28.0%) | 19 (27.5%) | 1.000 |
| AR | 327 (36.9%) | 105 (37.0%) | 1.000 | 91 (41.7%) | 17 (24.6%) | 0.016 |
| MR | 453 (51.1%) | 160 (56.3%) | 0.144 | 107 (49.1%) | 44 (63.8%) | 0.046 |
| TR | 320 (36.1%) | 110 (38.7%) | 0.469 | 85 (39.0%) | 27 (39.1%) | 1.000 |
| **Preoperative medications** | | | | | | |
| Nitrates | 36 (4.1%) | 13 (4.6%) | 0.837 | 6 (2.8%) | 4 (5.8%) | 0.263 |
| Catecholamine | 37 (4.2%) | 14 (4.9%) | 0.708 | 6 (2.8%) | 4 (5.8%) | 0.259 |
| Low molecular weight heparin | 252 (28.4%) | 87 (30.6%) | 0.527 | 56 (25.7%) | 26 (37.7%) | 0.077 |
| Metoprolol | 101 (11.4%) | 50 (17.6%) | 0.009 | 16 (7.3%) | 9 (13.0%) | 0.223 |
| ACEI | 22 (2.5%) | 7 (2.5%) | 1.000 | 9 (4.1%) | 7 (10.1%) | 0.080 |
| ARB | 26 (2.9%) | 14 (4.9%) | 0.155 | 4 (1.8%) | 4 (5.8%) | 0.093 |
| Statins | 80 (9.0%) | 40 (14.1%) | 0.020 | 14 (6.4%) | 11 (15.9%) | 0.028 |
| Non-statins | 25 (2.8%) | 15 (5.3%) | 0.072 | 4 (1.8%) | 3 (4.3%) | 0.373 |
| Aspirin | 62 (7.0%) | 32 (11.3%) | 0.029 | 14 (6.4%) | 10 (14.5%) | 0.063 |
| Clopidogrel | 45 (5.1%) | 26 (9.2%) | 0.018 | 12 (5.5%) | 7 (10.1%) | 0.263 |
| Ticagrelor | 27 (3.0%) | 9 (3.2%) | 1.000 | 1 (0.5%) | 5 (7.2%) | 0.007 |
| Hypoglycemic agents | 104 (11.7%) | 39 (13.7%) | 0.430 | 18 (8.3%) | 17 (24.6%) | 0.001 |
| Coenzyme complex | 136 (15.3%) | 54 (19.0%) | 0.172 | 38 (17.4%) | 15 (21.7%) | 0.531 |
| Argatroban | 18 (2.0%) | 7 (2.5%) | 0.839 | 3 (1.4%) | 1 (1.4%) | 1.000 |
| Sulodexide | 7 (0.8%) | 4 (1.4%) | 0.465 | 2 (0.9%) | 2 (2.9%) | 0.256 |
| Nicotinamide | 11 (1.2%) | 6 (2.1%) | 0.384 | 3 (1.4%) | 3 (4.3%) | 0.145 |
| **Surgical information** | | | | | | |
| Surgery type |  |  | <0.001 |  |  | 0.169 |
| CABG | 299 (33.7%) | 90 (31.7%) |  | 68 (31.2%) | 29 (42.0%) |  |
| Combined | 84 (9.5%) | 56 (19.7%) |  | 21 (9.6%) | 8 (11.6%) |  |
| Valve | 503 (56.8%) | 138 (48.6%) |  | 129 (59.2%) | 32 (46.4%) |  |
| CABG | 381 (43.0%) | 143 (50.4%) | 0.036 | 86 (39.4%) | 37 (53.6%) | 0.053 |
| Mitral valve surgery | 409 (46.2%) | 143 (50.4%) | 0.245 | 107 (49.1%) | 32 (46.4%) | 0.800 |
| Aortic valve surgery | 301 (34.0%) | 105 (37.0%) | 0.394 | 81 (37.2%) | 15 (21.7%) | 0.026 |
| Tricuspid valve surgery | 252 (28.4%) | 99 (34.9%) | 0.048 | 68 (31.2%) | 21 (30.4%) | 1.000 |
| Biological valve | 237 (26.7%) | 102 (35.9%) | 0.004 | 57 (26.1%) | 24 (34.8%) | 0.217 |
| Mechanical valve | 281 (31.7%) | 75 (26.4%) | 0.106 | 82 (37.6%) | 11 (15.9%) | 0.001 |
| RFTA | 71 (8.0%) | 28 (9.9%) | 0.395 | 29 (13.3%) | 6 (8.7%) | 0.419 |
| Left auricle surgery | 55 (6.2%) | 28 (9.9%) | 0.051 | 18 (8.3%) | 5 (7.2%) | 0.988 |
| Minimally invasive approach | 30 (3.4%) | 9 (3.2%) | 1.000 | 12 (5.5%) | 1 (1.4%) | 0.210 |
| **Intraoperative condition** | | | | | | |
| Medication propofol | 637 (71.9%) | 201 (70.8%) | 0.772 | 159 (72.9%) | 49 (71.0%) | 0.875 |
| Medication dexmedetomidine | 723 (81.6%) | 245 (86.3%) | 0.085 | 178 (81.7%) | 55 (79.7%) | 0.855 |
| Medication ulinastatin | 172 (19.4%) | 59 (20.8%) | 0.677 | 48 (22.0%) | 8 (11.6%) | 0.084 |
| Medication medrol | 7 (0.8%) | 7 (2.5%) | 0.030 | 1 (0.5%) | 3 (4.3%) | 0.048 |
| Medication hydrocortisone | 76 (8.6%) | 26 (9.2%) | 0.858 | 21 (9.6%) | 10 (14.5%) | 0.362 |
| PH | 7.4 (0.1) | 7.4 (0.1) | 0.040 | 7.4 (0.1) | 7.4 (0.1) | 0.187 |
| PaCO_2_, mmHg | 42.5 (28.0) | 41.7 (22.7) | 0.635 | 44.2 (37.9) | 45.4 (44.8) | 0.849 |
| PaO_2_, mmHg | 241.9 (59.4) | 236.4 (57.1) | 0.158 | 244.9 (59.3) | 240.3 (53.3) | 0.541 |
| BE, mmol/L | -1.7 (2.6) | -2.4 (2.8) | <0.001 | -1.3 (3.3) | -2.9 (3.1) | 0.001 |
| Hemoglobin, g/L | 8.1 (1.3) | 8.0 (1.3) | 0.052 | 8.2 (1.3) | 7.5 (1.2) | <0.001 |
| HCT, % | 24.3 (3.8) | 24.0 (3.9) | 0.214 | 24.5 (4.1) | 22.7 (4.4) | 0.004 |
| Blood products | 294 (33.2%) | 125 (44.0%) | 0.001 | 73 (33.5%) | 30 (43.5%) | 0.173 |
| RBC transfusion, units | 0.2 (0.9) | 0.6 (1.7) | 0.001 | 0.2 (0.8) | 0.4 (1.2) | 0.108 |
| Plasma transfusion, mL | 17.3 (88.7) | 53.0 (248.7) | 0.018 | 17.1 (76.9) | 35.5 (120.4) | 0.235 |
| CRYO transfusion, units | 1.0 (3.5) | 1.8 (4.9) | 0.007 | 0.6 (2.9) | 1.7 (4.5) | 0.052 |
| Platelet transfusion, units | 2.1 (4.7) | 2.6 (5.4) | 0.246 | 2.5 (4.7) | 2.6 (5.7) | 0.891 |
| IABP | 6 (0.7%) | 7 (2.5%) | 0.019 | 0 (0.0%) | 5 (7.2%) | <0.001 |
| **Cardiopulmonary bypass** | | | | | | |
| CPB time, min | 97.9 (36.2) | 111.5 (39.4) | <0.001 | 103.3 (37.8) | 103.9 (34.5) | 0.906 |
| ACC time, min | 67.4 (28.2) | 76.9 (30.8) | <0.001 | 71.4 (29.2) | 69.7 (29.5) | 0.671 |
| Assisted circulation time, min | 24.3 (11.0) | 27.1 (12.7) | 0.001 | 25.0 (10.6) | 27.6 (11.2) | 0.091 |
| Cardioplegia type | 851 (96.0%) | 272 (95.8%) | 0.975 | 210 (96.3%) | 67 (97.1%) | 1.000 |
| Cardioplegia temperature | 850 (95.9%) | 272 (95.8%) | 1.000 | 210 (96.3%) | 66 (95.7%) | 1.000 |
| Potassium cardioplegia | 669 (75.5%) | 193 (68.0%) | 0.015 | 158 (72.5%) | 47 (68.1%) | 0.585 |
| Venous cannula |  |  | 0.458 |  |  | 0.150 |
| Atria | 548 (61.9%) | 168 (59.2%) |  | 134 (61.5%) | 35 (50.7%) |  |
| Cavity | 338 (38.1%) | 116 (40.8%) |  | 84 (38.5%) | 34 (49.3%) |  |
| Arterial cannula |  |  | 1.000 |  |  | 0.157 |
| Aorta | 875 (98.8%) | 280 (98.6%) |  | 215 (98.6%) | 66 (95.7%) |  |
| Femoral | 11 (1.2%) | 4 (1.4%) |  | 3 (1.4%) | 3 (4.3%) |  |
| Myocardial perfusion volume | 819.0 (384.9) | 769.0 (387.4) | 0.059 | 794.5 (418.7) | 776.4 (373.9) | 0.734 |
| Myocardial perfusion time, min | 18.3 (32.6) | 20.9 (24.9) | 0.159 | 20.3 (27.2) | 19.1 (24.0) | 0.735 |
| Myocardial perfusion site |  |  | 1.000 |  |  | 0.225 |
| Coronary | 226 (25.5%) | 72 (25.4%) |  | 59 (27.1%) | 13 (18.8%) |  |
| Root | 660 (74.5%) | 212 (74.6%) |  | 159 (72.9%) | 56 (81.2%) |  |
| CUF, mL/kg | 18.2 (12.8) | 24.4 (15.2) | <0.001 | 19.2 (12.2) | 26.5 (18.4) | 0.003 |
| Urine output, mL/kg/hr | 4.5 (5.0) | 3.2 (3.0) | <0.001 | 4.2 (4.3) | 3.4 (3.2) | 0.102 |
| Spontaneous return | 631 (71.2%) | 206 (72.5%) | 0.725 | 161 (73.9%) | 52 (75.4%) | 0.927 |
| Defibrillate | 0.4 (0.9) | 0.4 (0.8) | 0.713 | 0.3 (0.7) | 0.2 (0.5) | 0.397 |
| Dosage of heparin | 51.5 (28.4) | 58.7 (33.5) | 0.001 | 55.0 (29.6) | 49.1 (28.3) | 0.141 |
| Nasopharyngeal temperature (℃) | 33.2 (0.9) | 33.0 (0.9) | 0.001 | 33.1 (0.9) | 33.2 (0.9) | 0.954 |
| Bladder temperature (℃) | 34.2 (1.0) | 34.0 (0.9) | 0.005 | 34.0 (1.0) | 34.0 (1.4) | 0.870 |
| Rectal temperature (℃) | 36.2 (0.4) | 36.2 (0.4) | 0.611 | 36.2 (0.4) | 36.2 (0.3) | 0.296 |
| ACT_T1, sec | 505.2 (85.6) | 503.3 (77.4) | 0.728 | 506.5 (91.1) | 527.8 (82.4) | 0.071 |
| ACT_T2, sec | 513.2 (89.5) | 506.2 (87.1) | 0.242 | 511.1 (86.2) | 533.5 (93.9) | 0.081 |
| ACT_T3, sec | 495.1 (72.8) | 491.9 (71.1) | 0.515 | 492.7 (74.2) | 504.7 (75.6) | 0.250 |
| Perfusion flow_T1, L/min/m^2^ | 2.6 (0.3) | 2.6 (0.3) | 0.395 | 2.6 (0.3) | 2.6 (0.3) | 0.917 |
| Perfusion flow_T2, L/min/m^2^ | 2.5 (0.5) | 2.6 (0.5) | 0.479 | 2.6 (0.4) | 2.5 (0.4) | 0.529 |
| Perfusion flow_T3, L/min/m^2^ | 1.6 (1.0) | 2.0 (0.9) | <0.001 | 1.6 (1.0) | 1.8 (0.9) | 0.103 |
| Perfusion pressure_T1, mmHg | 82.1 (7.4) | 81.7 (8.1) | 0.539 | 82.5 (8.0) | 80.0 (9.1) | 0.038 |
| Perfusion pressure_T2, mmHg | 65.2 (7.4) | 65.3 (6.8) | 0.812 | 64.6 (7.6) | 62.9 (7.5) | 0.109 |
| Perfusion pressure_T3, mmHg | 72.6 (8.9) | 72.8 (8.7) | 0.699 | 72.5 (8.3) | 71.3 (10.6) | 0.378 |
| Perfusion pressure_T4, mmHg | 82.2 (6.6) | 82.0 (6.3) | 0.692 | 82.6 (6.6) | 79.9 (7.3) | 0.008 |
| Oxygen flow_T1, L/min | 2.2 (0.4) | 2.2 (0.3) | 0.047 | 2.2 (0.3) | 2.2 (0.3) | 0.358 |
| Oxygen flow_T2, L/min | 2.1 (0.5) | 2.1 (0.4) | 0.475 | 2.2 (0.4) | 2.1 (0.4) | 0.055 |
| Oxygen flow_T3, L/min | 1.3 (0.8) | 1.7 (0.8) | <0.001 | 1.3 (0.8) | 1.6 (0.8) | 0.056 |
| CVP_T1, cmH_2_O | 6.5 (3.0) | 6.8 (2.9) | 0.163 | 6.6 (3.1) | 6.7 (3.2) | 0.922 |
| CVP_T2, cmH_2_O | 3.2 (3.2) | 3.8 (3.2) | 0.010 | 3.3 (3.0) | 3.9 (3.8) | 0.174 |
| CVP_T3, cmH_2_O | 5.2 (2.8) | 6.7 (3.3) | <0.001 | 5.0 (3.2) | 6.0 (3.7) | 0.059 |
| CVP_T4, cmH_2_O | 6.9 (3.0) | 8.8 (3.9) | <0.001 | 6.9 (3.1) | 8.1 (4.3) | 0.040 |
| **Early postoperative laboratory test** | | | | | | |
| PH | 7.4 (0.1) | 7.4 (0.1) | 0.099 | 7.4 (0.1) | 7.4 (0.1) | 0.325 |
| PaCO_2_, mmHg | 30.3 (5.3) | 30.8 (5.8) | 0.134 | 30.7 (5.8) | 30.4 (4.6) | 0.637 |
| Intubated PaO_2_/FiO_2_ ratio | 374.5 (102.6) | 332.5 (105.9) | <0.001 | 386.6 (105.7) | 318.9 (93.7) | <0.001 |
| Serum sodium, mmol/L | 139.7 (2.8) | 139.3 (3.2) | 0.115 | 139.5 (2.9) | 139.1 (3.5) | 0.297 |
| Serum potassium, mmol/L | 4.2 (0.5) | 4.5 (0.7) | <0.001 | 4.2 (0.5) | 4.5 (0.7) | <0.001 |
| Serum calcium, mmol/L | 1.2 (0.1) | 1.2 (0.1) | 0.168 | 1.2 (0.1) | 1.2 (0.1) | 0.515 |
| Lactic acid, mmol/L | 2.1 (1.3) | 2.2 (1.5) | 0.145 | 2.1 (1.2) | 2.3 (1.9) | 0.303 |
| BE, mmol/L | -4.1 (2.2) | -4.1 (2.8) | 0.822 | -4.0 (2.2) | -4.6 (2.2) | 0.084 |
| HCO_3_, mmol/L | 19.0 (2.3) | 19.1 (2.6) | 0.567 | 19.2 (2.3) | 18.8 (2.2) | 0.183 |
| WBC count (×10^^9^/L) | 13.3 (3.6) | 13.2 (4.1) | 0.937 | 13.1 (3.3) | 13.3 (4.7) | 0.814 |
| Lymphocyte count (×10^^9^/L) | 0.8 (0.4) | 0.8 (0.4) | 0.684 | 0.8 (0.4) | 0.8 (0.5) | 0.715 |
| Monocyte count (×10^^9^/L) | 0.9 (0.4) | 0.9 (0.4) | 0.832 | 0.8 (0.4) | 0.9 (0.5) | 0.550 |
| Neutrophil count (×10^^9^/L) | 11.6 (3.3) | 11.6 (3.7) | 0.920 | 11.5 (3.0) | 11.6 (4.1) | 0.814 |
| NLR | 18.0 (9.4) | 18.8 (11.8) | 0.324 | 17.6 (9.8) | 20.3 (16.6) | 0.200 |
| PLR | 231.4 (132.9) | 238.2 (174.2) | 0.547 | 233.0 (128.4) | 242.3 (145.5) | 0.637 |
| LMR | 1.1 (0.7) | 1.1 (0.8) | 0.954 | 1.3 (1.4) | 1.1 (1.1) | 0.399 |
| RBC count (×10^^12^/L) | 3.5 (0.5) | 3.4 (0.5) | <0.001 | 3.5 (0.5) | 3.3 (0.4) | 0.001 |
| Hemoglobin, g/L | 107.9 (14.1) | 99.8 (13.4) | <0.001 | 107.9 (13.7) | 98.2 (11.4) | <0.001 |
| HCT, % | 32.0 (4.2) | 30.7 (4.6) | <0.001 | 31.9 (4.0) | 29.9 (3.5) | <0.001 |
| RDW, % | 13.5 (1.4) | 14.0 (1.8) | <0.001 | 13.7 (1.5) | 14.0 (1.3) | 0.091 |
| Platelet count (×10^^9^/L) | 147.5 (50.4) | 144.4 (50.7) | 0.367 | 154.9 (56.2) | 144.6 (53.7) | 0.175 |
| MPV, fL | 11.5 (1.1) | 11.6 (1.1) | 0.448 | 11.5 (1.2) | 11.4 (1.2) | 0.488 |
| PDW, % | 14.5 (2.5) | 14.6 (2.3) | 0.484 | 14.4 (2.6) | 14.3 (2.5) | 0.828 |
| ALT, U/L | 34.4 (35.3) | 34.8 (55.7) | 0.908 | 39.4 (47.8) | 27.5 (20.2) | 0.004 |
| AST, U/L | 43.2 (39.7) | 50.0 (59.3) | 0.072 | 53.2 (53.4) | 41.7 (29.4) | 0.024 |
| LDH, U/L | 347.0 (99.3) | 398.7 (133.4) | <0.001 | 349.5 (95.5) | 381.2 (112.1) | 0.036 |
| Albumin, g/L | 32.1 (4.5) | 32.3 (4.9) | 0.389 | 32.0 (4.3) | 31.8 (4.6) | 0.726 |
| Bilirubin, g/L | 16.6 (9.2) | 16.9 (10.7) | 0.687 | 17.5 (9.8) | 14.9 (9.4) | 0.051 |
| TBA, μmol/L | 0.9 (2.6) | 1.4 (2.3) | 0.005 | 0.8 (1.1) | 1.4 (3.3) | 0.146 |
| PT, sec | 11.9 (1.1) | 12.0 (1.3) | 0.501 | 11.9 (1.2) | 12.1 (1.9) | 0.455 |
| INR | 1.0 (0.1) | 1.0 (0.1) | 0.319 | 1.0 (0.1) | 1.1 (0.2) | 0.276 |
| APTT, sec | 30.3 (7.5) | 31.0 (7.3) | 0.165 | 30.6 (6.3) | 31.6 (16.7) | 0.614 |
| Fibrinogen, g/L | 2.7 (0.9) | 2.8 (0.8) | 0.169 | 2.7 (1.2) | 2.7 (0.7) | 0.820 |
| D-dimer, μg/mL | 1.1 (1.2) | 1.2 (1.8) | 0.647 | 1.2 (2.2) | 1.1 (1.2) | 0.739 |

Data are presented as means (standard deviation) or number (%). The clinical characteristics of patients who developed AKI or not were compared using the t-test, Mann-Whitney U-test, chi-square test, or Fisher’s exact probability method

**Table S2.** Five-fold cross-validation results of AUROC and accuracy of XGBoost, random forest, and deep forest model

| **Classifiers** | **AUROC** | **Accuracy** |
| --- | --- | --- |
| XGBoost | | |
| Fold-1 | 0.797 | 0.825 |
| Fold-2 | 0.808 | 0.818 |
| Fold-3 | 0.788 | 0.787 |
| Fold-4 | 0.820 | 0.819 |
| Fold-5 | 0.858 | 0.825 |
| Average | 0.814 | 0.815 |
| Random forest | | |
| Fold-1 | 0.819 | 0.825 |
| Fold-2 | 0.804 | 0.808 |
| Fold-3 | 0.821 | 0.804 |
| Fold-4 | 0.843 | 0.814 |
| Fold-5 | 0.873 | 0.833 |
| Average | 0.832 | 0.817 |
| Deep forest | | |
| Fold-1 | 0.848 | 0.830 |
| Fold-2 | 0.901 | 0.853 |
| Fold-3 | 0.839 | 0.846 |
| Fold-4 | 0.802 | 0.827 |
| Fold-5 | 0.884 | 0.863 |
| Average | 0.855 | 0.843 |

AUROC, area under the receiver operating characteristic curve

**Table S3.** Classifiers’ predictive performance in the validation set

| **Classifiers** | **Sensitivity (%)** | **Specificity (%)** | **PPV (%)** | **NPV (%)** | **F score** |
| --- | --- | --- | --- | --- | --- |
| Logistic regression | 43.5 | 95.4 | 75.0 | 84.2 | 0.83 |
| XGBoost | 53.6 | 95.4 | 78.7 | 86.7 | 0.85 |
| Random forest | 52.2 | 96.8 | 83.7 | 86.5 | 0.86 |
| Deep forest | 59.4 | 94.5 | 77.4 | 88.0 | 0.86 |

PPV, positive predictive value; NPV, negative predictive value
